# Cardiomyocytes Sense Matrix Rigidity through a Combination of Muscle and Non-muscle Myosin Contractions

**DOI:** 10.1016/j.devcel.2018.05.016

**Published:** 2018-06-04

**Authors:** Pragati Pandey, William Hawkes, Junquiang Hu, William Valentine Megone, Julien Gautrot, Narayana Anilkumar, Min Zhang, Liisa Hirvonen, Susan Cox, Elisabeth Ehler, James Hone, Michael Sheetz, Thomas Iskratsch

## Main Text

(Developmental Cell *44*, 326–336; February 5, 2018)

In the originally published version of this paper, a set of brackets was inadvertently left off of Equation 3 in the STAR Methods section during the preparation of the manuscript. The calculations in the paper were not affected by this error, which has now been corrected in the article online. The authors apologize for any confusion that this error may have caused.

