# Supplementary material for: Cardiomyocytes Sense Matrix Rigidity through a Combination of Muscle and Non-muscle Myosin Contractions
Source: Dev Cell. 2018 Feb 5;44(3):326–336.e3. doi: 10.1016/j.devcel.2017.12.024 (PMC5807060; doi:10.1016/j.devcel.2017.12.024)
Supplement: Document S1. Figures S1–S9 [file mmc1.pdf]

Developmental Cell, Volume *44*

Supplemental Information

Cardiomyocytes Sense Matrix Rigidity through a Combination of Muscle and Non-muscle Myosin Contractions

Pragati Pandey, William Hawkes, Junquiang Hu, William Valentine Megone, Julien Gautrot, Narayana Anilkumar, Min Zhang, Liisa Hirvonen, Susan Cox, Elisabeth Ehler, James Hone, Michael Sheetz, and Thomas Iskratsch

Supplementary Information

Figure S1

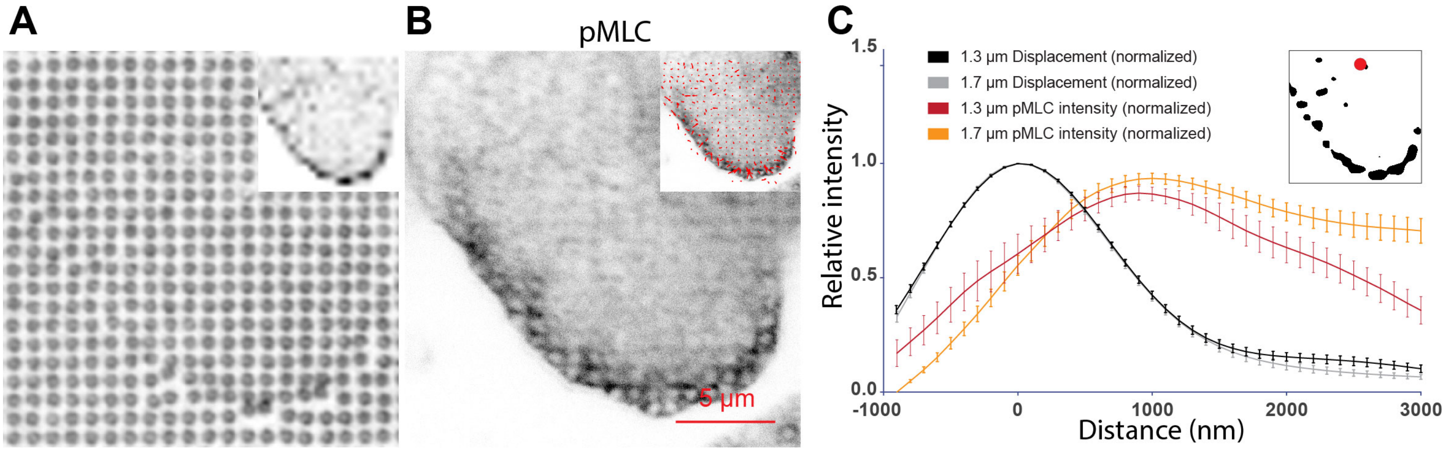

Figure S1: Non-muscle myosin light chain is active close to force transduction sites. Freshly isolated NRC were cultured on quantum dot labelled PDMS pillars (A, inset shows the grey scale coded displacement map) for 48 hours, fixed and stained for pMLC T18/S19 (B, overlaid with displacement vectors in inset). C) Radial profiles were calculated from displacements that were higher than the 90th percentile (inset, black areas) to the centre of the cell (inset, marked with red dot). Error bars: SEM; N=12 cells. Related to Figure 1&3.

Figure S2

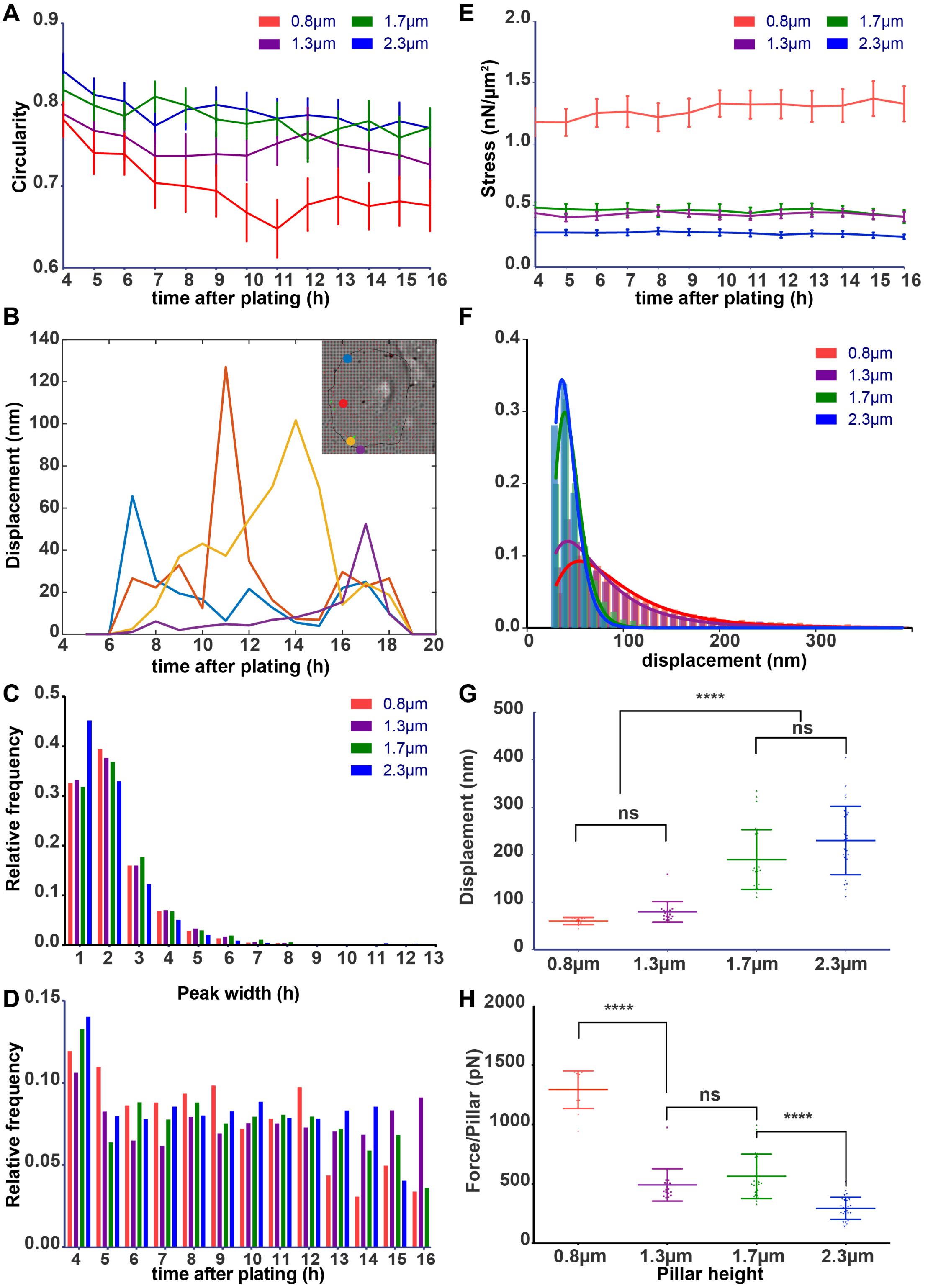

Figure S2 Analysis of spreading on Pillars of different stiffness. A) A reduction in circularity is observed during spreading, but faster and more pronounced on stiff pillars. B) Examples of individual pillar displacement traces for pillars indicated in the inset. C) Length of displacement events (i.e. peak width) shows no difference between pillar dimensions. D) Time of occurrence of the displacement peaks suggests that there is continued probing of the environment on all pillar dimensions. E) Because the cell is only applying the tension at the cell edge, there is no change of the stress over time. F) Pillar displacements follow a log-normal distribution. G-H) As expected for a log-normal distribution, the sharp change in displacement at equal force between the 1.3 µm and 1.7 µm high pillars is also detected when analysing the 90th percentile for each cell. Error bars: SEM (A,E) or mean ± SD (G,H); * p<0.05; ** p<0.01; *** p<0.001; **** p<0.0001; ns: not significant; P values from ANOVA and Tukey correction for multiple comparisons. Related to Figure 1.

Figure S3

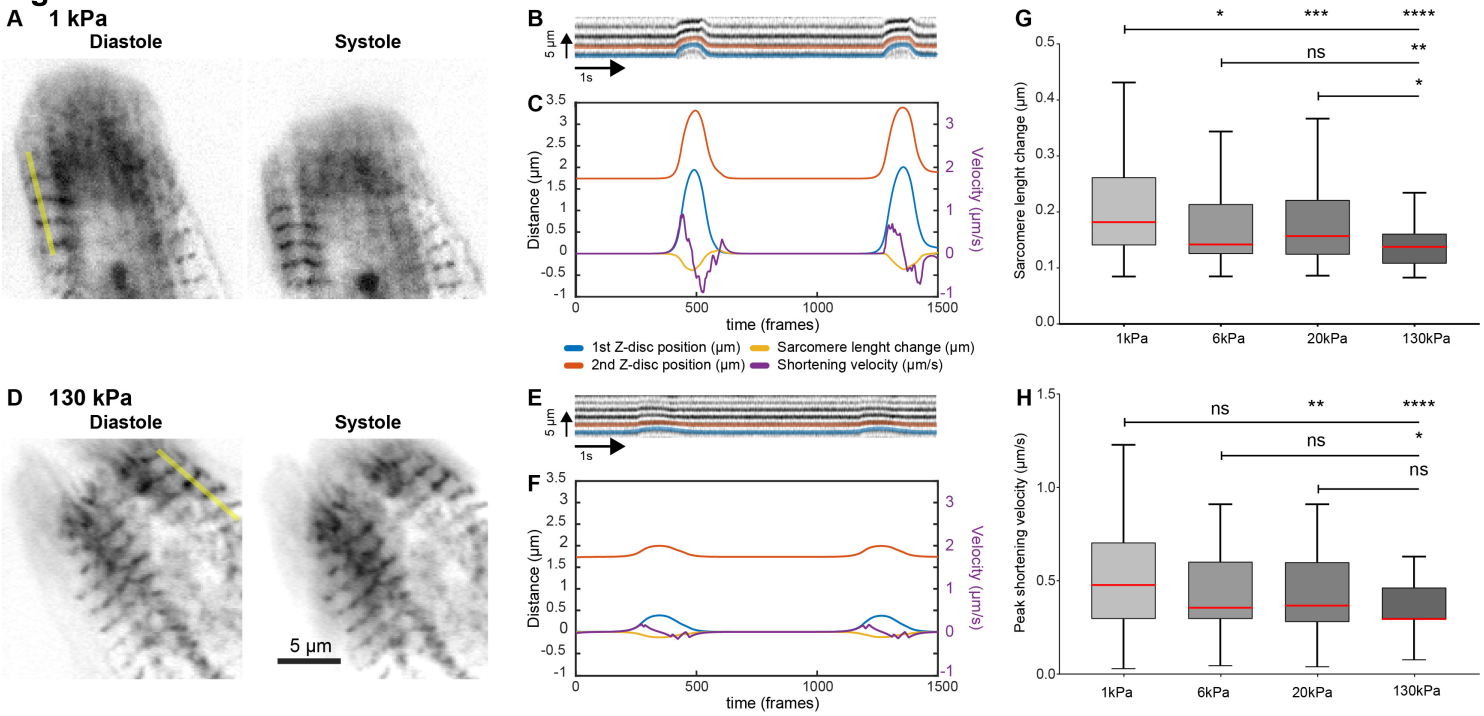

Figure S3 Contraction velocity is higher on soft PDMS. A) High speed movies (209 fps) of contracting, α-actinin GFP adenovirus infected neonatal cardiomyocytes were taken on PDMS surfaces with 1, 6, 20 and 130 kPa stiffness. A,D) Snapshots from cells during diastole and systole on 1 and 130 kPa respectively. B,E) Kymographs were drawn and used to measure z-disc positions during the contraction cycle and calculate sarcomeric length change and shortening velocity(C,F; A-C: 1kPa; D-E: 130kPa). G) Sarcomeres shorten to a larger extent on soft surfaces. H) Peak shortening velocity is higher on soft surfaces. n=11, 12, 11 and 13 cells for 1, 6, 20 and 130 kPa respectively. The first 4 sarcomeres from the cell edge were shortening to equal extent (data not shown) and hence were combined for the statistics in G and H.

* $p<0.05$; ** $p<0.01$; *** $p<0.001$; **** $p<0.0001$; ns: not significant; P values from ANOVA and Tukey correction for multiple comparisons. Related to Figure 1.

Figure S4

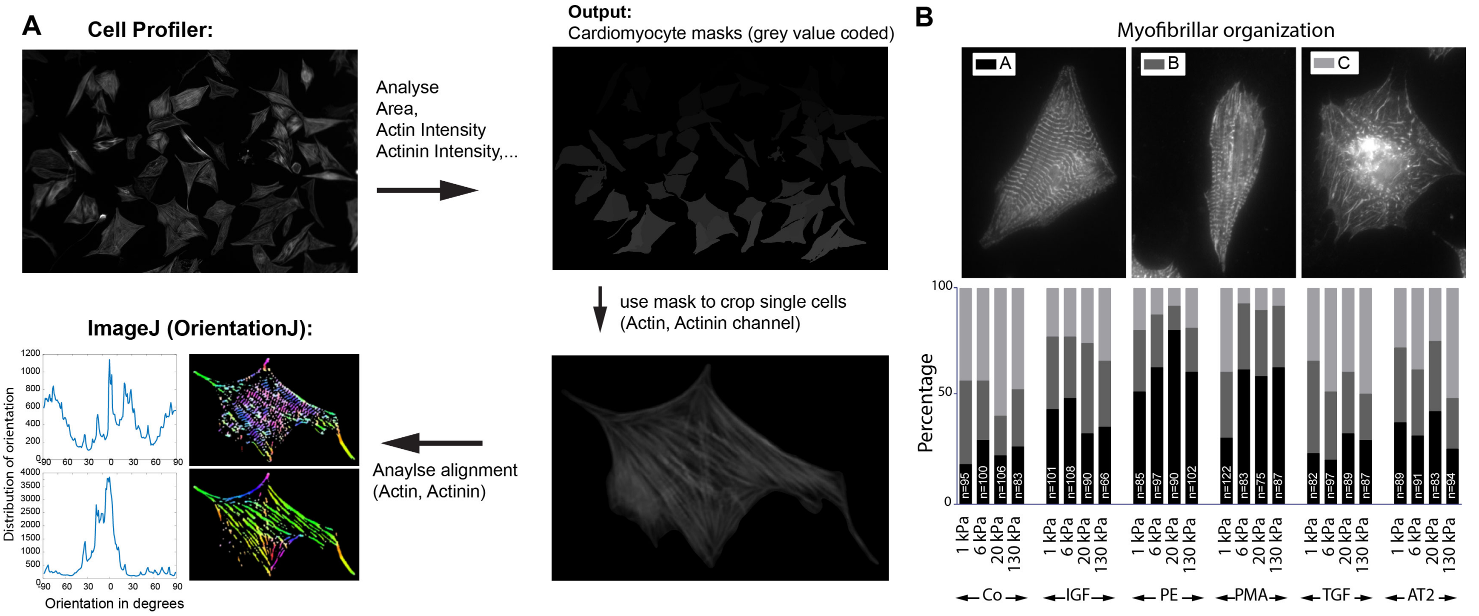

Figure S4 Analysis pipeline for multiwell assays. A) Images of NRC stained with α-actinin, Phalloidin and DAPI were taken on a large format CMOS camera (4908*3264px). Dapi and Phalloidin staining were used to segment cells in cell profiler and cardiomyocytes were selected based on α-actinin staining intensity. Cells were analysed for cell area, shape and staining intensities and a grey scale mask image was created as output image, based on which single cells were cropped. Images of single cells were then analysed with the orientationJ plugin in imageJ. Colours in the output images indicate orientation and saturation the coherency. Myofibrillar maturation results in higher anisotropy and fewer orientation peaks, with higher coherency values. The maximum value was aligned to 0 degrees, and averaged for all cells as indicator of the level of alignment. The result was confirmed manually by categorizing cells into three categories as displayed above (A: Mature myofibrils; B: mix of mature myofibrils with stress fibre-like structures (SFLS); C: mostly SFLS). N-numbers as indicated in the graph. Related to Figure 2.

Figure S5

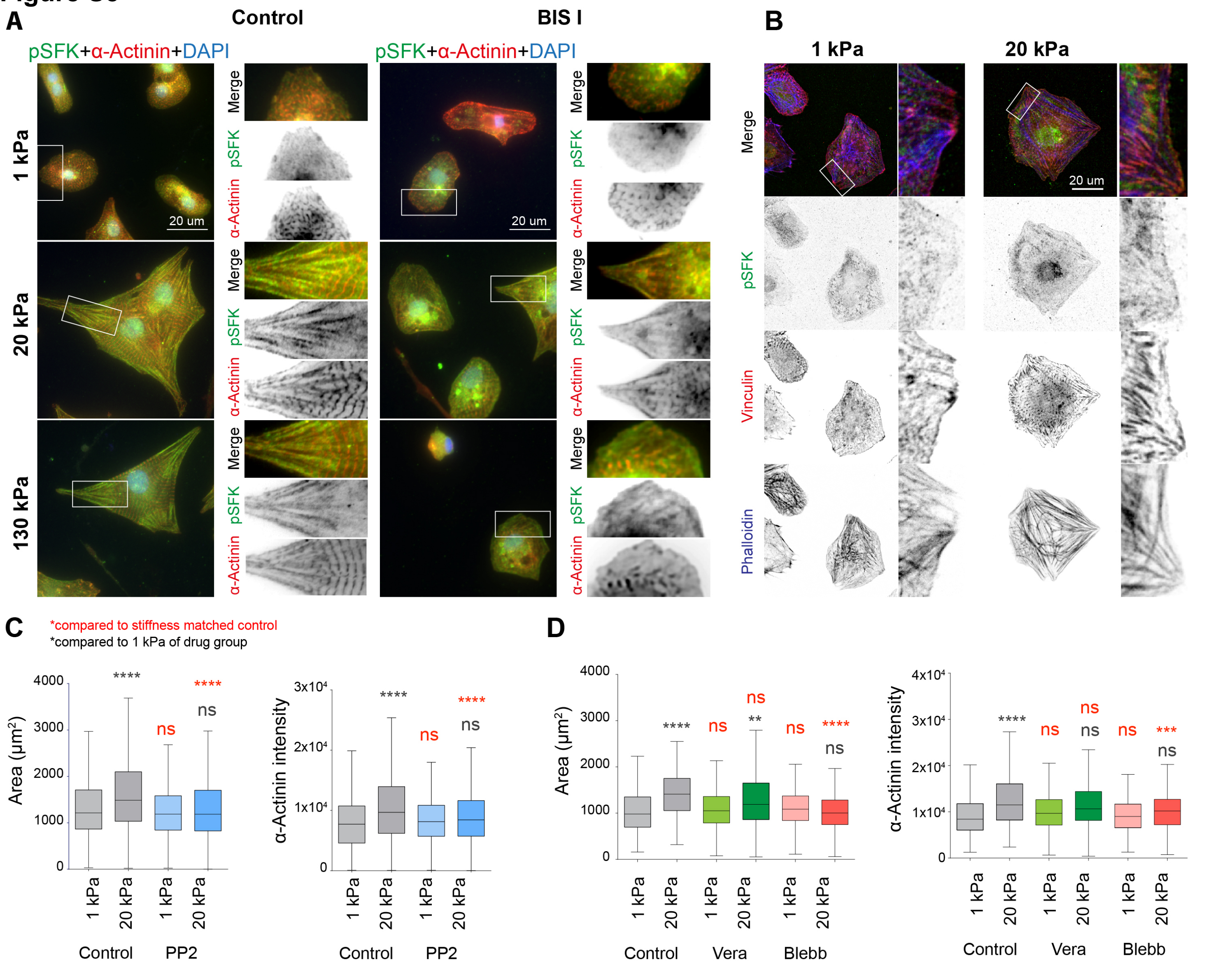

Figure S5: Src is activated downstream of PKC and required for cardiomyocyte rigidity sensing A) NRCs cultured on PDMS coverslips were stained for pSFK and α-actinin. Treatment with BIS I reduces the activity of active Src at the cell edge. B) During cardiomyocyte spreading the pSFK staining partially overlaps with Vinculin on stiff, but not on soft surfaces. C) Treatment with PP2 reduces the rigidity dependent differences in cell area and α-actinin staining intensity. D) The rigidity dependence of cell area and α-actinin staining intensity is reduced slightly after verapamil treatment (Vera) and abolished after blebbistatin treatment (Blebb). Error bars: SEM; N>100 cells for all conditions; * $p<0.05$; ** $p<0.01$; *** $p<0.001$; **** $p<0.0001$; ns: not significant; P values from ANOVA and Tukey correction for multiple comparisons. Related to Figure 3.

Figure S6

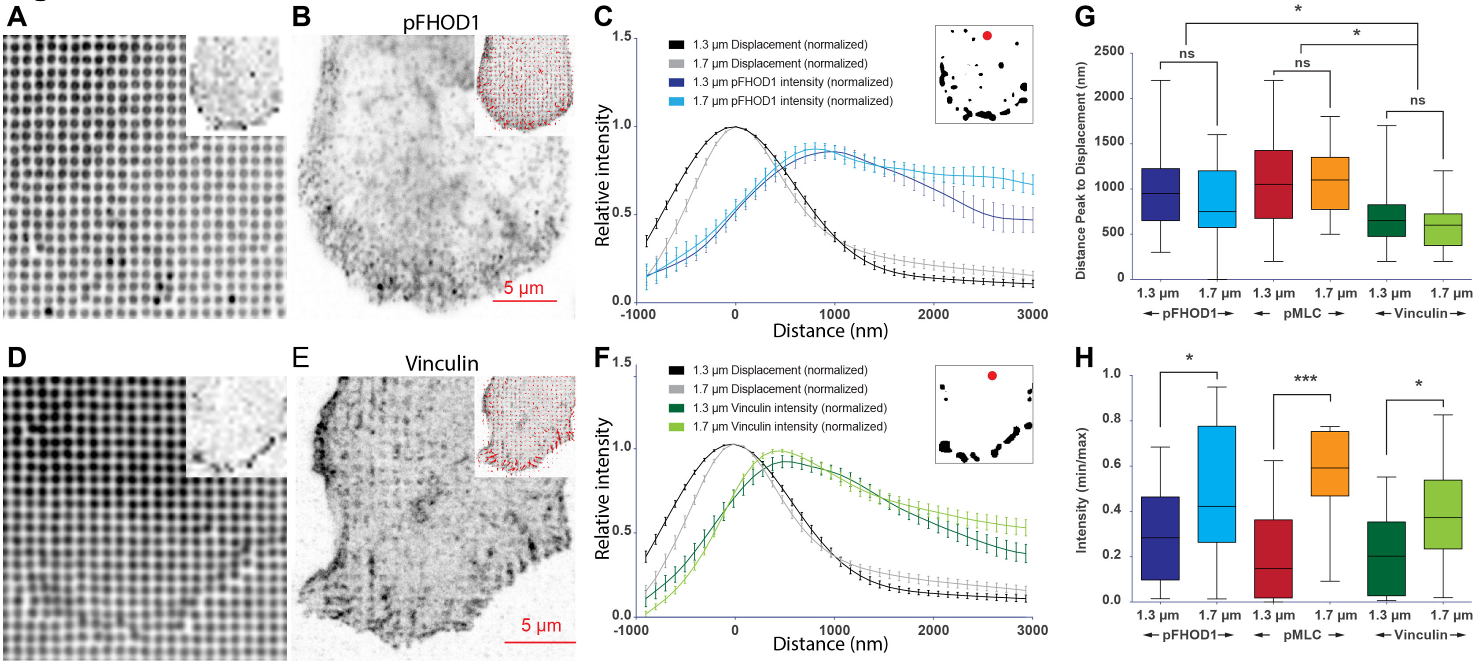

Figure S6: Vinculin and pFHOD1 localize to sites of large pillar displacements. A-F) Freshly isolated NRC were cultured on quantum dot labelled PDMS pillars (A,D, inset shows the grey scale coded displacement map) for 48 hours, fixed and stained for pFHOD1 (B), or Vinculin (E); insets show displacement staining with overlaid displacement vectors. C,F) Radial profiles were calculated from displacements that were higher than the 90th percentile (inset, black areas) to the centre of the cell (inset, marked with red dot). G) All three proteins are enriched inwards of displaced pillars, but Vinculin peaks closer to the displaced pillars. For pMLC, see also Figure S1. H) pFHOD1, pMLC (see Figure S1) and Vinculin are more strongly enriched at force transduction sites on 1.3 µm than on 1.7 µm pillars. Error bars: SEM; Box plots: Tukey; n>12 cells for all conditions. * p<0.05; ** p<0.01; *** p<0.001; **** p<0.0001; ns: not significant; P values from ANOVA and Tukey correction for multiple comparisons. Related to Figure 3.

Figure S7:

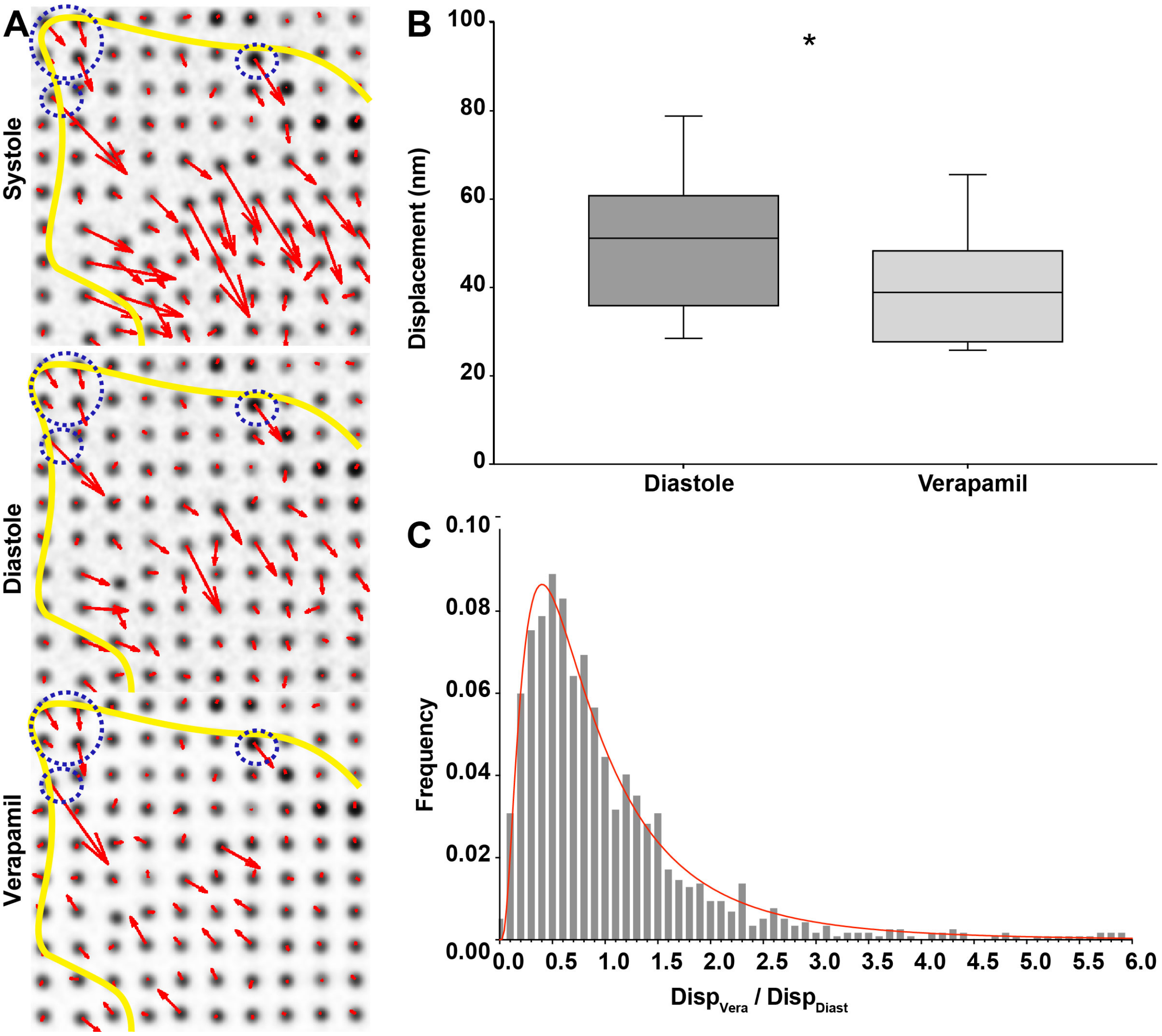

Figure S7: Comparison of diastolic resting tension and residual tension after verapamil treatment. A) Freshly isolated NRC were cultured on quantum dot labelled PDMS pillars (1.3 µm height, k=6pN/nm). Movies were recorded of contracting cardiomyocytes to extract pillar positions during systole and diastole (A), before treatment with verapamil (10µM) for 10 minutes. Further images of the cell were taken after visual confirmation that contractions were blocked (A, Verapamil), and after trypsinisation to recover the original pillar positions. B) Comparison of average pillar displacements of n=12 cells per condition shows a 15% reduction after verapamil treatment (*: p=0.03 from a ratio paired t-test). C) Pillar by pillar analysis of changes to the displacement, combined for all 12 cells. The ratio of the displacement after verapamil treatment vs the displacement during diastole shows a lognormal distribution, peaking around 0.6, i.e. a 40% loss of tension. Related to Figure 3.

Figure S8

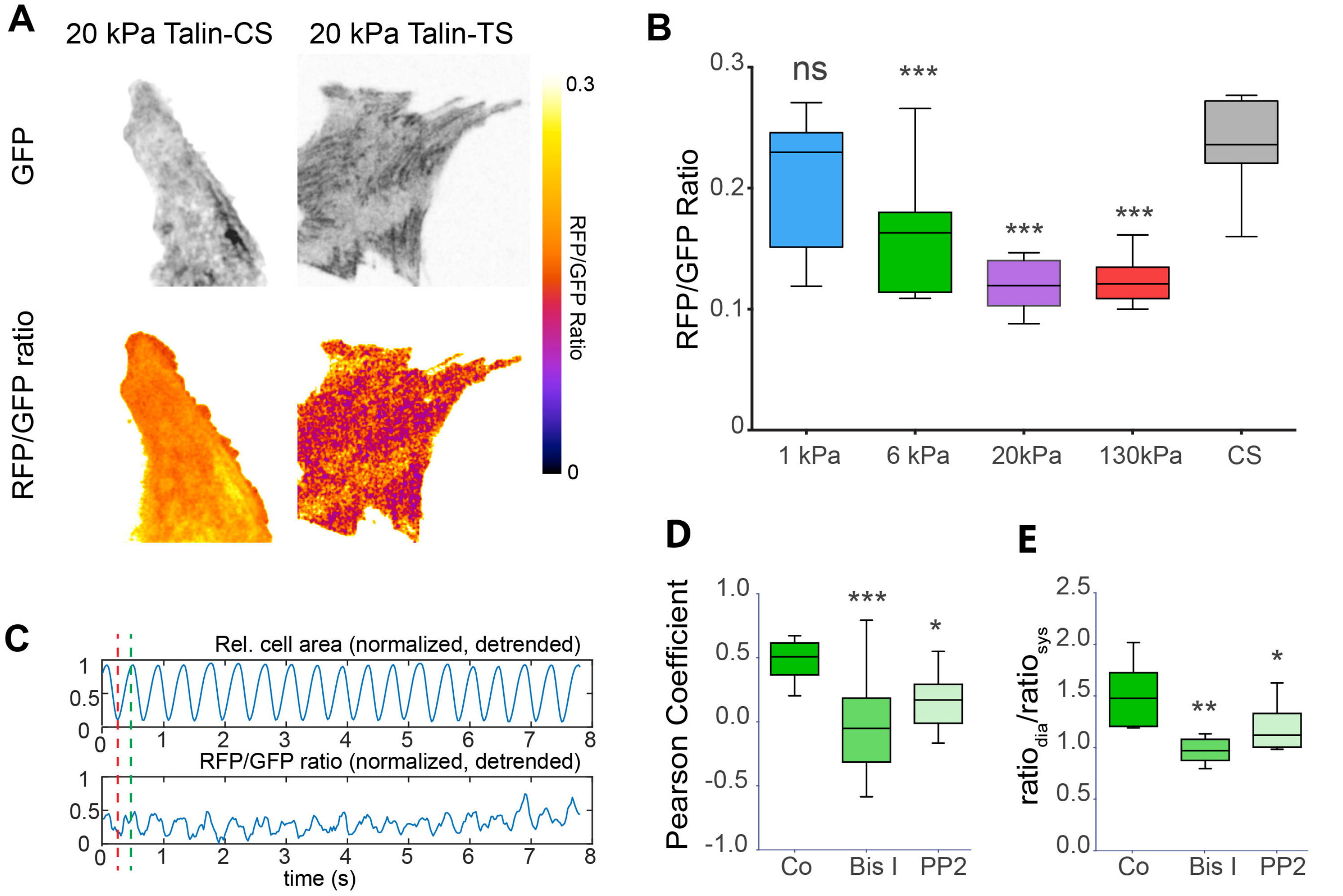

Figure S8: The talin tension sensor indicates higher tension on stiff surfaces in fixed cells. A-B) Freshly isolated NRC, transfected with the Talin tension sensor (TS) or control sensor (CS) were cultured on PDMS coated coverslips for 48 hours, stained and imaged on a ZEISS LSM 880 microscope with spectral detector after illumination with a 488nm laser. The RFP to GFP ratio was calculated after linear unmixing. C) Live cells were imaged with 40 frames per second. The cell edge position was tracked after thresholding and compared to the RFP/GFP ratio at adhesion sites (see Figure 4B), after normalization and detrending to remove changes due to differential bleaching of the fluorophores. Example traces for the cell area and ratio are shown in (C). The Pearson correlation coefficient was calculated (summarized in Figure 4C) and minima (red line) as well as maxima (green line) were identified in the cell area plot and ratio values were averaged over all minima or maxima, respectively (summarized in Figure 4D). Treatment with Bis I or PP2 reduces both Pearson coefficient (D) and the FRET ratio between diastole and systole (E). N=12 cells; Box plot: Tukey; * $p<0.05$; ** $p<0.01$; *** $p<0.001$; **** $p<0.0001$; ns: not significant; P values from ANOVA and Tukey correction for multiple comparisons. Related to Figure 4.

Figure S9

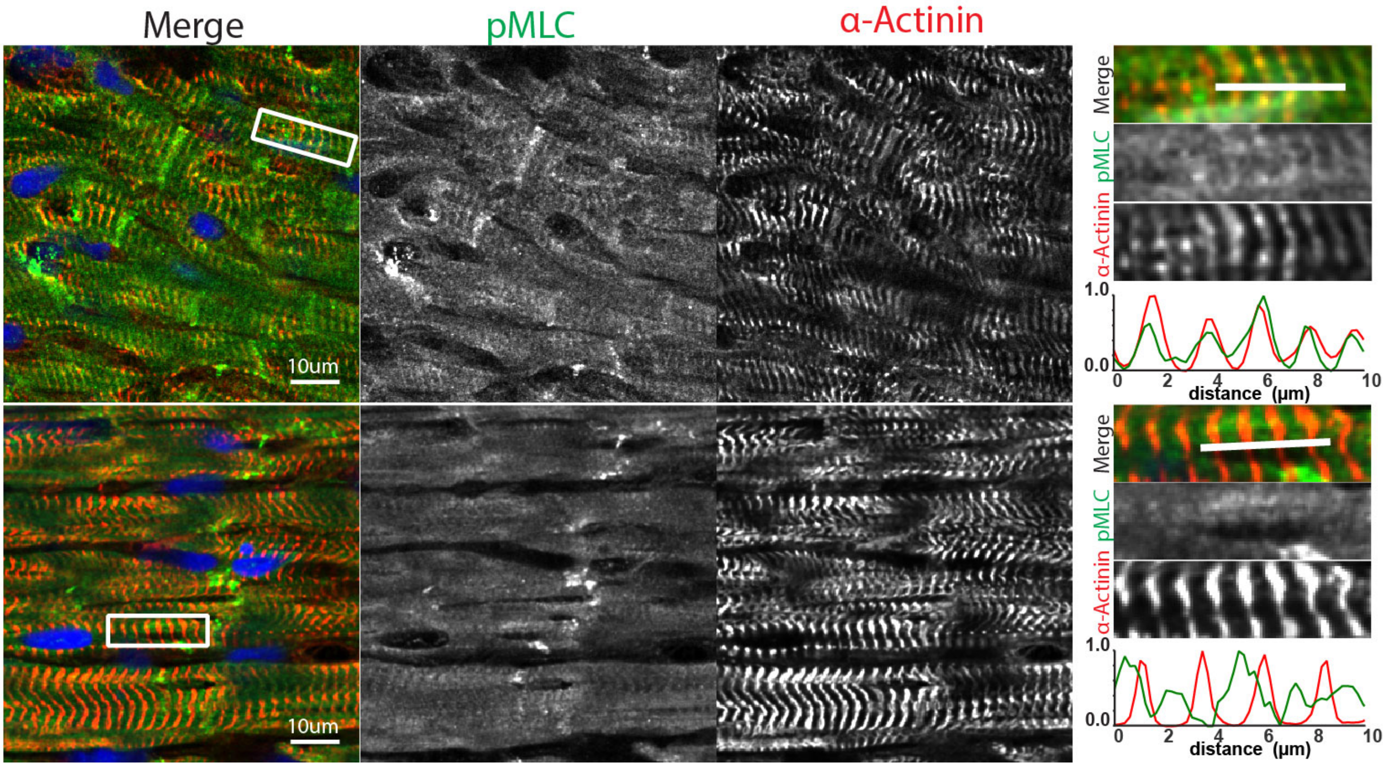

Figure S9: Elevated non-muscle myosin activity in dilate cardiomyopathy. pMLC is found in striations that partially overlap with α-actinin in hearts of the MLP-KO mouse, but not in wild type controls. Related to Figure 5.
