# Supplementary material for: Cardiomyocytes Sense Matrix Rigidity through a Combination of Muscle and Non-muscle Myosin Contractions
Source: Dev Cell. 2018 Feb 5;44(3):326–336.e3. doi: 10.1016/j.devcel.2017.12.024 (PMC5807060; doi:10.1016/j.devcel.2017.12.024)
Supplement: Document S2. Article plus Supplemental Information [file mmc6.pdf]

Article

Developmental Cell

Cardiomyocytes Sense Matrix Rigidity through a Combination of Muscle and Non-muscle Myosin Contractions

Graphical Abstract

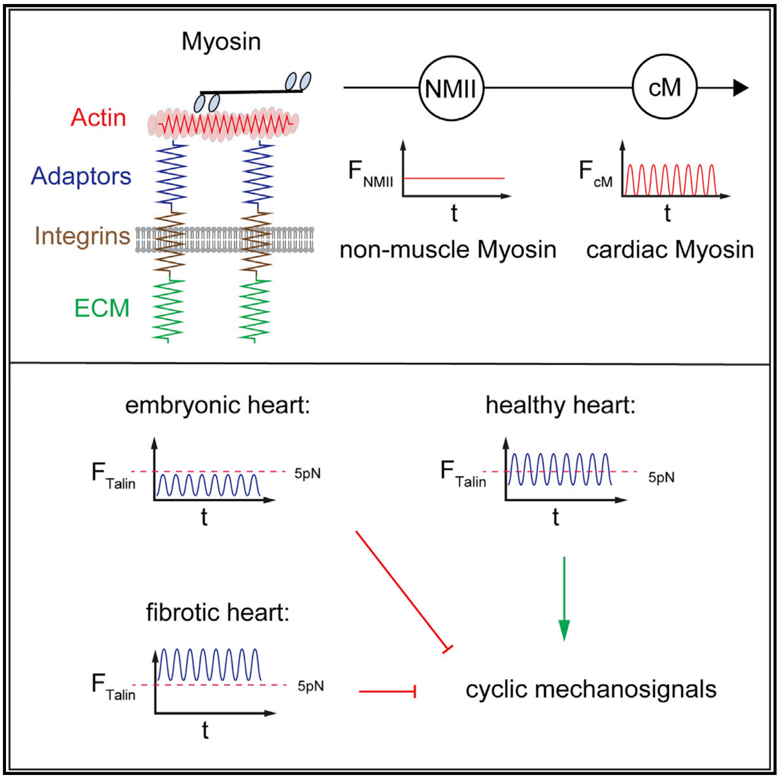

Highlights

- Talin in cardiomyocytes is unstretched, cyclically stretched, or continuously stretched
- Talin stretching depends on stiffness, myofibrillar tension, and non-myofibrillar tension
- Non-myofibrillar contractility requires PKC, Src, FHOD1, and non-muscle myosin
- PKC and non-muscle myosin activity are enhanced in cardiac disease

Authors

Pragati Pandey, William Hawkes, Junquiang Hu, ..., James Hone, Michael Sheetz, Thomas Iskratsch

Correspondence

t.iskratsch@qmul.ac.uk

In Brief

Pandey et al. identify that cardiomyocytes sense the rigidity of the heart by measuring the combined forces from non-muscle and muscle myosin. This can result in cyclic or continuous stretching of the mechanosensitive protein talin, depending on the substrate stiffness and the level of non-muscle myosin activity.

Pandey et al., 2018, Developmental Cell *44*, 326–336
February 5, 2018 © 2018 The Authors. Published by Elsevier Inc.
https://doi.org/10.1016/j.devcel.2017.12.024

CellPress

Developmental Cell
Article

Cardiomyocytes Sense Matrix Rigidity through a Combination of Muscle and Non-muscle Myosin Contractions

Pragati Pandey,[1,2] William Hawkes,[1,2] Junquiang Hu,[3] William Valentine Megone,[2] Julien Gautrot,[2] Narayana Anilkumar,[4] Min Zhang,[4] Liisa Hirvonen,[1] Susan Cox,[1] Elisabeth Ehler,[1,4] James Hone,[3] Michael Sheetz,[5,6] and Thomas Iskratsch[1,2,7,*]
[1]Randall Centre for Cell and Molecular Biophysics, King's College London, SE1 1UL London, UK
[2]Institute of Bioengineering and School of Engineering and Materials Science, Queen Mary University of London, E1 4NS London, UK
[3]Department of Mechanical Engineering, Columbia University, New York 10027, USA
[4]School of Cardiovascular Medicine and Sciences, King's College London, SE5 9NU London, UK
[5]Department of Biological Sciences, Columbia University, New York 10027, USA
[6]Mechanobiology Institute, National University of Singapore, Singapore 117411, Singapore
[7]Lead Contact
*Correspondence: t.iskratsch@qmul.ac.uk
https://doi.org/10.1016/j.devcel.2017.12.024

SUMMARY

Mechanical properties are cues for many biological processes in health or disease. In the heart, changes to the extracellular matrix composition and cross-linking result in stiffening of the cellular microenvironment during development. Moreover, myocardial infarction and cardiomyopathies lead to fibrosis and a stiffer environment, affecting cardiomyocyte behavior. Here, we identify that single cardiomyocyte adhesions sense simultaneous (fast oscillating) cardiac and (slow) non-muscle myosin contractions. Together, these lead to oscillating tension on the mechanosensitive adaptor protein talin on substrates with a stiffness of healthy adult heart tissue, compared with no tension on embryonic heart stiffness and continuous stretching on fibrotic stiffness. Moreover, we show that activation of PKC leads to the induction of cardiomyocyte hypertrophy in a stiffness-dependent way, through activation of non-muscle myosin. Finally, PKC and non-muscle myosin are upregulated at the costameres in heart disease, indicating aberrant mechanosensing as a contributing factor to long-term remodeling and heart failure.

INTRODUCTION

Cells in the myocardium are exposed to different types of forces, including hemodynamic pressure, stretching forces through the cardiomyocyte contraction, as well as passive elasticity from the extracellular matrix. All of these are subject to change during development and disease. In particular, the composition of the extracellular matrix shows spatial and temporal changes during cardiac development, including differential expression of matrix proteases, proteoglycans, glycosaminoglycans, and glycoproteins, such as collagens, fibronectin, or laminin (Rienks et al., 2014; Lockhart et al., 2011). Together these changes result in an increase of cardiac stiffness from the low kilopascal range in the embryonic heart to ~10 kPa in the adult heart (during diastole) (Huyer et al., 2015; Majkut et al., 2013; Jacot et al., 2010). On the other hand, cardiac fibrosis results in increased expression and cross-linking of extracellular matrix components, thereby again changing the mechanical landscape, and rigidities above 100 kPa have been reported for fibrotic tissue (Wei et al., 2015; Szibor et al., 2014; Jacot et al., 2010; Soufen et al., 2008; Bishop and Laurent, 1995). Over the past decade there has been accumulating evidence that a wide range of different cell types can probe and react to changes in rigidity (Iskratsch et al., 2014). Also, several studies have investigated the role of matrix stiffness in primary or stem cell derived cardiomyocytes. While the contractile stress increases with the elastic modulus of the matrix, myofibrillar maturity and contractile work show a biphasic response and are highest on ~10 kPa hydrogels (McCain et al., 2014; Hersch et al., 2013; Hazeltine et al., 2012; Rodriguez et al., 2011; Engler et al., 2008; Jacot et al., 2008). This indicates that the expression of myofibrillar proteins, as well as the assembly and maturation of myofibrils, respond to stiffness. However, it is still unknown how cardiomyocytes measure the rigidity to influence gene expression pattern or myofibrillogenesis.

To investigate this, we use here nanopillars, which have been an invaluable tool for mechanotransduction studies in various cell types, mainly because of their high spatial and temporal resolution, compared with conventional traction force microscopy (Meacci et al., 2016; Wolfenson et al., 2016; Tabdanov et al., 2015; Iskratsch et al., 2014; Iskratsch et al., 2013b). Also, in contrast with micropillars, they have no obvious effects on cell morphology or adhesion formation (Ghassemi et al., 2012). For our study, we combine nanopillars with flat silicon gels with defined rigidity as a screening platform and for complementary experiments, including Förster resonance energy transfer (FRET) tension sensor measurements. In this way, we identify that cardiomyocytes sense a combination of slow non-muscle myosin and fast muscle myosin contractions that result in

 © 2018 The Authors. Published by Elsevier Inc.
This is an open access article under the CC BY license (http://creativecommons.org/licenses/by/4.0/).

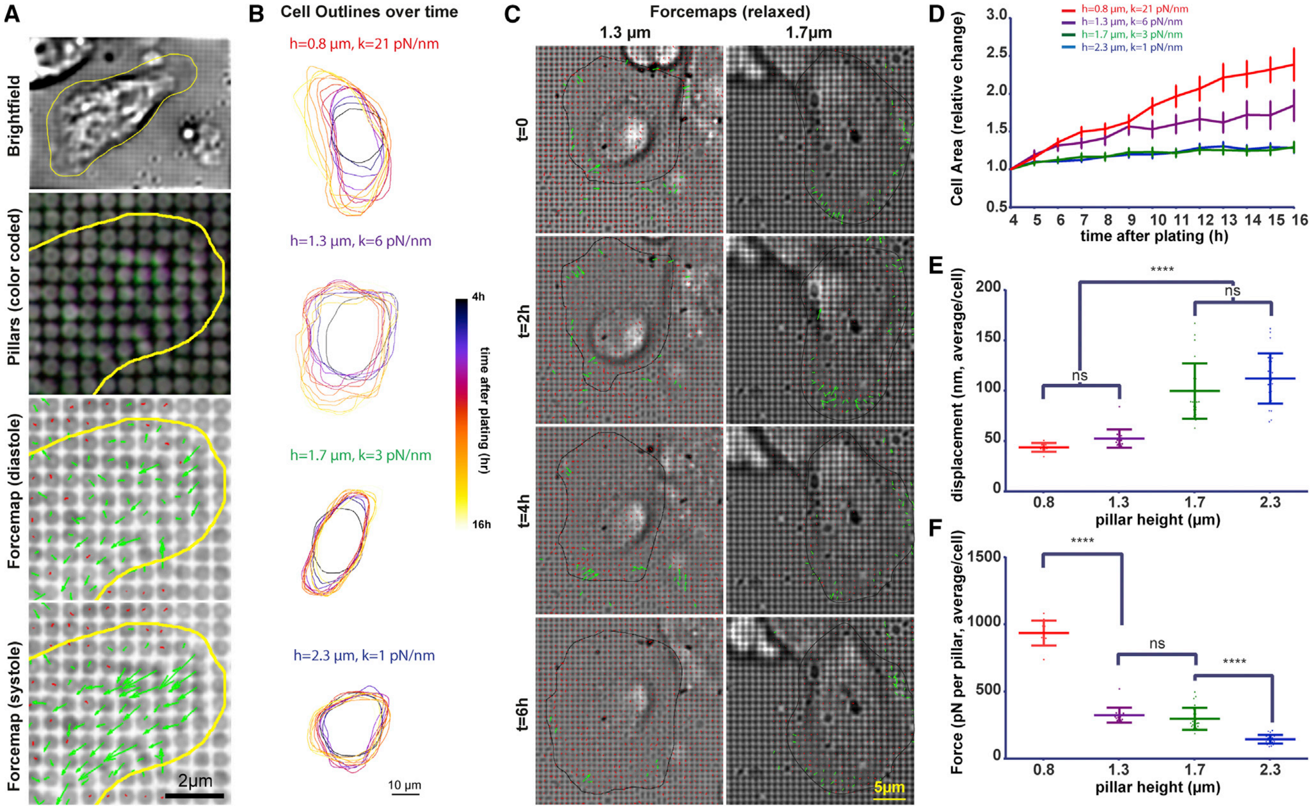

Figure 1. NRC Spreading on Pillars Indicates Repeated Probing of Environment

Freshly isolated neonatal rat cardiomyocytes (NRCs) were plated on PDMS pillars and imaged overnight every hour, after an initial spreading period (4 hr).

(A) To distinguish between systole and diastole, three consecutive frames, with 200 ms delay, were taken at each time point and cells in diastole were stacked together.

(B) Example cell outlines, color coded for each time point as indicated in the color lookup table.

(C) Example force maps for NRCs on 1.3- or 1.7-µm-tall pillars indicate continuous probing of the environment.

(D) NRCs spread faster on stiff pillar surfaces (0.8 and 1.3 µm). Error bars: SEM.

(E and F) Analysis of pillar displacements (E, N > 100 pillars per cell and >12 cells per condition, displayed as average per cell) indicate a big jump in pillar displacements between the 1.3 and 1.7 µm pillars whereby the force remains constant in this stiffness range (F). Error bars: mean ± SD (E, F). ****$p < 0.0001$; ns, not significant; p values from ANOVA and Tukey correction for multiple comparisons.

See also Figures S1–S3; Movies S1, S2, and S3.

oscillating stretching of the mechanosensitive protein talin on physiological substrate rigidities but continuous stretching on fibrotic stiffness. Moreover, we find that activation of PKC results in alteration of rigidity sensing via the modulation of non-muscle myosin contractility. Importantly, upregulation of PKC and non-muscle myosin activity is also evident in disease models for myocardial infarction (MI) and dilated cardiomyopathy even in non-fibrotic areas. Therefore, we find that cardiomyocyte mechanosignaling is affected in heart disease through altered stiffness, as well as humoral factors.

RESULTS

Cardiomyocytes Use Non-myofibrillar Contractions to Apply Tension on the Environment during Spreading

Previous studies have highlighted the effect of extracellular matrix stiffness on the myofibrillar organization and contractile properties. To identify how cardiomyocytes sense the mechanical properties of their environment, we first plated freshly isolated neonatal rat cardiomyocytes (NRCs) on fibronectin coated, quantum dot labeled polydimethylsiloxane (PDMS) nanopillar arrays with a stiffness of 1, 3, 6, and 21 pN/nm (or a calculated equivalent elastic modulus of 4, 10, 22, and 78 kPa) and followed them during spreading. Initial observations indicated the presence of spontaneous contractions, typically lasting for ~200 ms, and additional forces at the cell edge with slower dynamics, overlapping with a staining for active non-muscle myosin light chain (further referred to here as non-myofibrillar contractions; Figure S1 and Movie S1). To analyze the magnitude and localization of the non-myofibrillar forces during spreading, we took three subsequent frames (separated by 200 ms) of the quantum dot labeled pillars at each time point and stacked together images of the cells in diastole (Figure 1A). Comparable with previous studies, we found larger cell areas, lower circularity, and faster spreading on stiffer matrices (Figures 1B, 1D, and S2A). Non-myofibrillar forces were limited to the cell edge and the individual pillars were pulled by the cardiomyocytes for durations between 1 and 2 hr on all pillar dimensions

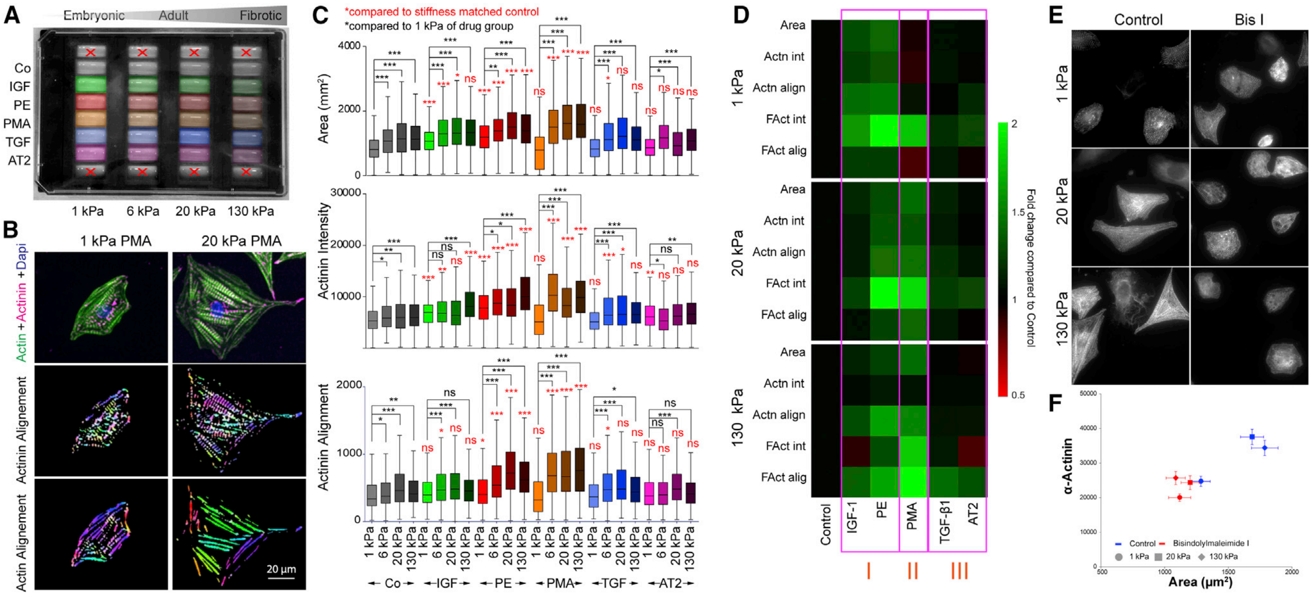

Figure 2. Multi-rigidity Assay to Identify Inducers of Rigidity Sensing

(A) NRCs were plated on a multiwell plate with four different rigidities, serum starved, and treated with IGF-1, phenylephrine (PE), PMA, TGFβ1, or angiotensin II (AT2) for 48 hr.

(B and C) Cells were stained with phalloidin and α-actinin (B) and analyzed with cell profiler (size, shape, intensity) and ImageJ (alignment, see also Figure S3) (C). Boxplot: Tukey.

(D) Depending on the response on different rigidities the stimuli can be grouped into those that act independent of rigidity (I), upstream of rigidity sensing (II), or show no significant change over control (III).

(E and F) PKC inhibition with BIS I results in cell areas (F) and α-actinin staining (E) comparable with control cells cultured on soft surfaces. N > 240 cells from three repeats for all conditions.

Error bars: SD. *p < 0.05; **p < 0.01; ***p < 0.001; ns, not significant; p values from ANOVA and Tukey correction for multiple comparisons. See also Figure S4.

(Figures 1C, S2B–S2D and Movies S2 and S3). This indicated that cells were continuously probing their environment, but the speed of cell area expansion depended on the stiffness. Because the forces were only applied at the cell edge, there was no overall increase in traction stress (Figure S2E). Analysis of the pillar displacements and resulting forces showed a non-linear relationship between stiffness and displacement with a big jump between 3 and 6 pN/nm pillar stiffness, equivalent to elastic moduli between 10 and 22 kPa (Figures 1E, 1F, and S2F–S2H) and approximately the range of rigidities that were previously reported for healthy adult heart tissue (Huyer et al., 2015; Majkut et al., 2013; Jacot et al., 2010). This surprising result indicated an intrinsic regulation of force generation or signaling that is pivotal for cardiomyocyte rigidity sensing.

PKC Activation Induces Stiffness-Dependent Cardiomyocyte Maturation

In fibrotic heart tissue, cardiomyocytes are exposed to abnormal matrix stiffness as well as cytokine and neurohumoral stimuli, some of which can also activate pathways that induce expansion of the cell area and the myofibrillar network in cardiomyocytes in culture (insulin growth factor 1 [IGF-1], transforming growth factor β1 [TGF-β1]). Similarly, activation of cardiomyocyte hypertrophy was reported after treatment with adrenergic agonists or after phorbol-ester-mediated activation of PKC isozymes (Watkins et al., 2012; Munoz et al., 2009; Vijayan et al., 2004; Braz et al., 2002; Schultz Jel et al., 2002; Taylor et al., 2000).

We aimed to identify reagents that activate myofibril formation in a rigidity-dependent way to allow controlled induction of rigidity sensing and detailed study of downstream effects with relevance for the *in vivo* situation. For this we plated cardiomyocytes on flat PDMS surfaces with defined stiffness, covering the stiffness range from the embryonic to the fibrotic heart stiffness (1, 6, 20, and 130 kPa; Figure 2A). To test the suitability of the surfaces for cardiomyocyte culture, we first measured contractile properties in high-speed movies (>200 frames per second [fps]) using GFP-tagged α-actinin as a marker for the Z-disc positions, from which we then extracted the extent and velocity of sarcomeric shortening (Figure S3). As expected, cells were contracting to a larger extent on soft surfaces (Figures S3A–S3G). Moreover, sarcomeres shortened faster on soft PDMS (Figure S3H), in agreement with a load/velocity relationship typical for muscle (Hill, 1938). Having confirmed the functionality of the cardiomyocytes on all stiffnesses, we next plated NRCs on multi-rigidity multiwell plates, serum starved the cells, and treated them with a range of reagents (phenylephrine [PE], angiotensin [AT], phorbol 12-myristate 13-acetate [PMA], IGF-1, TGF-β1) that were previously reported to induce cardiomyocyte hypertrophy (Figure 2A) (Watkins et al., 2012; Munoz et al., 2009; Vijayan et al., 2004; Braz et al., 2002; Schultz Jel et al., 2002; Taylor et al., 2000). After 48 hr of treatment, cells were fixed; stained for α-actinin and F-actin; and analyzed for cell area, staining intensity, and myofibril alignment (Figures 2A–2D and S4). Using this approach, we could identify reagents that were inducing

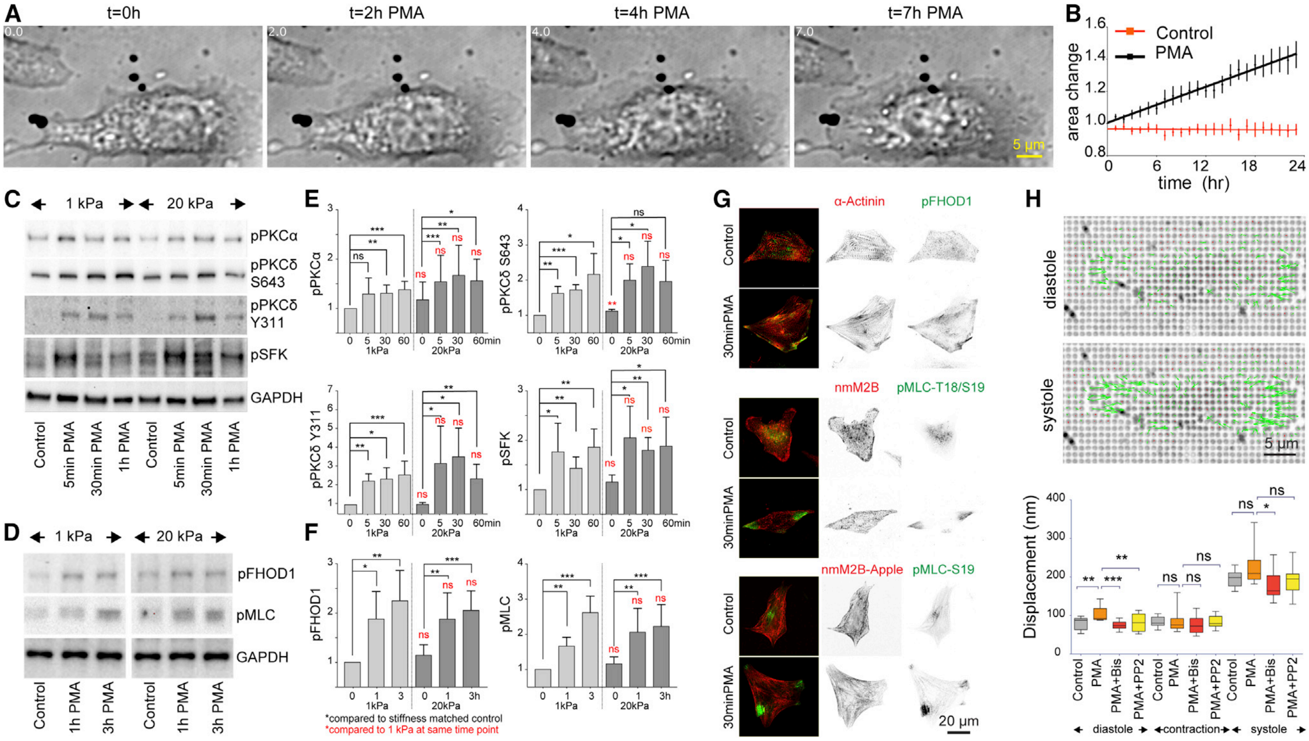

Figure 3. Activation of Src, Non-muscle Myosin, and FHOD1 after PMA Treatment

(A and B) PMA treatment activates cell expansion for several hours. (A) Frames from a bright field movie of an NRC on stiff pillars, whereby pillars were filtered out with a bandpass filter during post processing to enhance visibility of the cell. See also Movie S4. (B) Quantification of relative area change of n = 20 cardiomyocytes each (PMA treated and serum-starved control) from differential interference contrast movies on fibronectin coated coverslip dishes.

(C–G) PKCα and PKCδ are activated after PMA treatment. SFK and PKCδ Y311 phosphorylation, associated with increased activity, are strongly enhanced (C); quantified in (E) from n = 5, 3, 4, and 4 repeats for PKCα, PKCδ Ser643, PKCδ Y311, and pSFK, respectively. p values from unpaired t tests. (D) PMA treatment results in higher levels of FHOD1 and non-muscle myosin activity; quantified in (F) from n = 4 repeats. (G) PMA treatment also enhances localization of active FHOD1 (D, top) and non-muscle myosin light chain (D, middle, bottom) to the cell edge. Cells were counter stained with anti-α-actinin (top), anti-non-muscle myosin 2B (middle), or transfected with non-muscle myosin 2B-mApple.

(H) Cardiomyocytes were filmed at 10 fps on 1.7-μm-high pillars (k = 3 pN/nm) and pillar displacements were analyzed in diastole and peak systole. PMA treatment increases tension in diastole but not contractile forces. Diastolic tension returns to baseline levels after BIS I or PP2 treatment to inhibit PKC or Src. N > 13 cells with >100 pillars analyzed per cell for all conditions. Boxplot: Tukey.

*p < 0.05; **p < 0.01; ***p < 0.001; ns, not significant; p values from ANOVA and Tukey correction for multiple comparisons. See also Figures S5–S7; Movie S4.

cardiomyocyte hypertrophy independently of stiffness (PE, IGF-1), only on stiff (PMA), or on neither stiff nor soft surfaces (AT, TGF-β1) (Figures 2D and S4B). Because PMA was the only reagent inducing cardiomyocyte hypertrophy in a stiffness-dependent way and thus acting upstream of rigidity sensing, we next tested the effect of PKC inhibition with bisindolylmaleimide (BIS) II and I on cardiomyocyte phenotypes on different surfaces. Indeed, both BIS II (not shown) and BIS I abolished rigidity-dependent differences in cardiomyocyte phenotypes. The cell morphology and α-actinin staining intensity in BIS I-treated cells on soft and stiff surfaces were comparable with control cells on soft surfaces, thus confirming an involvement of PKC in cardiomyocyte rigidity sensing (Figures 2E and 2F).

PMA Induces Activation of Non-myofibrillar Contractility and Rapid Cell Area Expansion on Stiff Surfaces

We next analyzed the immediate response of cardiomyocytes to PMA treatment. On stiff surfaces, NRCs immediately formed protrusions and expanded the cell surface area over the time course of observation (Figures 3A and 3B and Movie S4). Western blot analysis indicated activation of PKCα and PKCδ (Figures 3C and 3E). It is noteworthy that, in line with previous reports (Miranti et al., 1999), we found a reduction in PKCα and phospho-PKC (pan and α/β1) levels after 3 hr and 24 hr (data not shown), indicating that PKC activation was the initial trigger but was dispensable for the long-term effects.

PKC is known to translocate to the membrane after integrin activation and PMA treatment, where it is activated. At the cell edge, its targets include several focal adhesion proteins as well as the non-receptor tyrosine kinase Src (through PTPα) (Brandt et al., 2003; Keenan and Kelleher, 1998). In agreement with this, we also detected rapid phosphorylation of PKCδ at an Src-dependent phosphorylation site (Y311), which is associated with the regulation of activity as well as substrate specificity and indicated a feedback mechanism (Rybin et al., 2008). This feedback mechanism was confirmed when we probed for changes to activity of Src depending on stiffness and PMA treatment. Here we found strong activation of Src and/or related kinases with a phospho-Src family kinase antibody (SFK) after

PMA treatment (Figure 3C). Moreover, during cardiomyocyte spreading, we found localization of active Src close to focal adhesions, where it was partially overlapping with α-actinin and vinculin staining pattern on stiff, but not soft, surfaces (Figures S5A and S5B). The localization of active Src close to the cell edge was lost after BIS treatment, confirming activation of Src downstream of PKC (Figure S5A). Stiffness-dependent differences in cell area and α-actinin staining intensity were abolished after Src inhibition with PP2 in agreement with a role for Src during cardiomyocyte rigidity sensing (Figure S5C).

It is noteworthy that, on the western blots, we did not detect stiffness-dependent differences in PKCα or Src activation in steady state (after serum starvation) or after PMA treatment and only a slightly higher PKCδ activity on stiff compared with soft surfaces in the serum-starved controls (Figure 3E). Therefore, our data show that PMA treatment leads to full activation of PKC on all stiffnesses, suggesting that rigidity sensing occurs downstream of PKC and Src.

Src activates actin assembly by regulating the localization and downstream activation of FHOD1, which is a prominent formin family protein in cardiomyocytes (Al Haj et al., 2015; Dwyer et al., 2014; Iskratsch et al., 2013b). Moreover, it is known to regulate activation of non-muscle myosin light chain through modulating the activity of ROCKII and MLCK (Shen et al., 2015; Lee et al., 2010; Birukov et al., 2001). Non-muscle myosin IIB heavy chain was present in stress-fiber-like structures in cardiomyocytes before and after PMA treatment (Figure 3G, middle, anti-nmM2B; bottom, transfected with nmM2B-apple). However, we found elevated levels of phosphorylated (active) non-muscle myosin (pMLC T18/S19), as well as FHOD1, after PMA treatment (Figures 3D and 3F) and increased activity of these proteins at the cell edge (pMLC T18/S19 and pMLC S19 as well as pFHOD1; Figures 3G, S1, and S6). Treatment of cardiomyocytes with the L-type calcium channel blocker verapamil immediately stopped myofibrillar contractions, but rigidity-dependent differences in cell area and α-actinin staining intensity were only slightly reduced. Blebbistatin, in contrast, completely abolished the differences, indicating that non-myofibrillar contractility is required for rigidity sensing (Figure S5D).

Therefore, we hypothesized that Src can activate non-muscle myosin force generation, especially at the cell edge, to regulate rigidity sensing. To test this, we took movies of contracting NRCs (10 fps) on PDMS pillars (k = 3 pN/nm) after 30 min of PMA treatment to resolve the myofibrillar contraction (Figure 3H). From the data we extracted the displacement during diastole, relative difference between systole and diastole, as well as peak systolic displacement, compared with the pillars in the original position after removal of the cells with trypsin. Here we found a significant increase in diastolic pillar displacements after PMA treatment that returned to the baseline level after concomitant treatment with BIS I or PP2, but we found no difference in the myofibrillar contraction strength itself (Figure 3H). Acute treatment with verapamil reduced the resting tension by ~15% (Figures S7A and S7B). Because verapamil blocks L-type (which are responsible for excitation-contraction coupling) as well as T-type channels (which are expressed in neonatal but not adult ventricular cardiomyocytes and fine-tune basal calcium levels) (Leuranguer et al., 2000; Nargeot, 2000; Sipido et al., 1998; Cai et al., 1997; Enyeart et al., 1993), the reduction in resting tension was likely due to altered diastolic calcium levels downstream of the latter. This therefore indicates a contribution from Ca^{2+}-sensitive elements, such as the titin N2B or PEVK domains, to the tension observed on the pillars (Fujita et al., 2004; Lahmers et al., 2004; Labeit et al., 2003; Linke et al., 2002). Also, in support of a role for titin, the tension was reduced to the largest extent on pillars that were contracted during systole, suggesting these were indeed myofibrillar attachment sites (Figure S7A).

Nevertheless, large contributions to the diastolic tension were insensitive to the verapamil treatment. This was especially the case at sites close to the cell edge (Figure S7A, blue circles), where we found the highest activity of non-muscle myosin (Figures 3G and S1). Indeed, both active pFHOD1 and pMLC were located close to sites of large pillar displacements (peaking about 1 μm inward of the original pillar center) and significantly more enriched on stiff than on soft pillars (Figures S1 and S6). Therefore, our data indicate that PKC enhances the non-myofibrillar contractility at the cell edge through activation of Src, FHOD1, and non-muscle myosin.

Talin Is Cyclically Stretched in Cardiomyocytes on Physiological Stiffness but Continuously Stretched on Fibrotic Stiffness

Because our data indicated a modification of the force production at the cell edge, we proceeded to a detailed analysis of cardiomyocyte forces using nanopillar arrays with different dimensions. In diastole, we again detected a sharp jump in pillar displacements between 6 and 3 pN/nm pillar stiffness from 47 ± 4 to 104 ± 21 nm, while the force remained constant (291 ± 27 versus 311 ± 60 pN, mean ± SD; Figure 4A). A similar jump in displacement between 6 and 3 pN/nm pillar stiffness was observed during systole (from 119 ± 13 to 229 ± 51 nm) but, unlike during diastole, we detected a further increase of displacements on the softest pillars (275 ± 47 nm on 1 pN/nm stiff pillars).

The displacement of the pillars is a function of the exerted forces (in this case myofibrillar and non-myofibrillar); the stiffness of the pillars; as well as the stiffness of the integrins and adaptors and the breakage force of the respective bonds (Roca-Cusachs et al., 2012). Thereby the softest element will experience the greatest strain (inversely proportional to its stiffness compared with the stiffness of the other elements). Thus, our data suggested that while on 1 pN/nm pillars the pillars will be deformed to the largest extent, mechanosensitive adaptor proteins such as talin (Roca-Cusachs et al., 2012; del Rio et al., 2009) could be deformed proportionally and therefore buffer a part of the load (Elosegui-Artola et al., 2014). Effects of adhesion reinforcement will further enhance the integrin-cytoskeleton bond strength and enable higher forces on high stiffness (Elosegui-Artola et al., 2014; Chan and Odde, 2008).

Compared with non-muscle adhesions, which experience force with a single loading rate from attached actin stress fibers, we find that cardiomyocyte adhesions experience overlapping slow non-myofibrillar forces and fast myofibrillar forces that result in pillar displacements of >1 μm/s. Therefore, our data could indicate proportionally stronger stretching of mechanosensitive adaptor proteins during the myofibrillar contractions. Several mechanosensitive proteins have been reported so far, including titin, p130Cas, vinculin, and talin (Zhou et al., 2015;

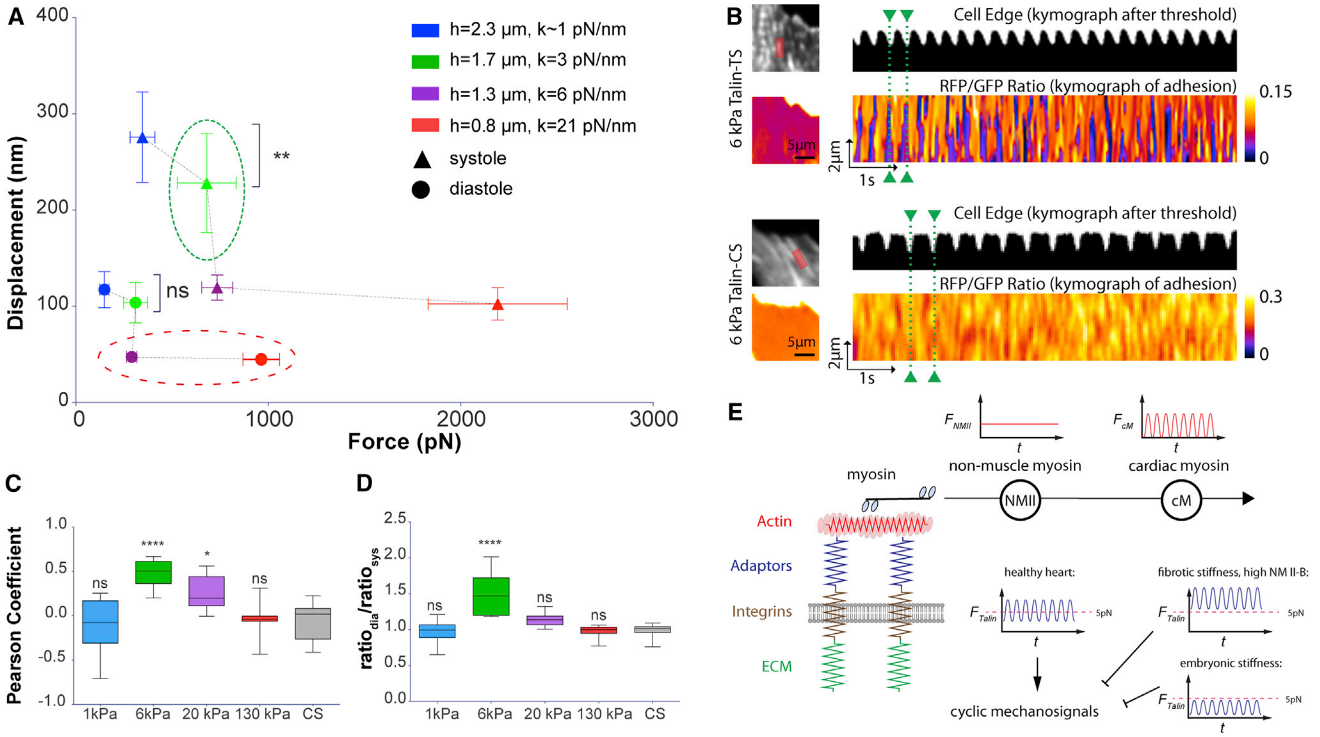

Figure 4. Mechanosensitive Proteins Are Cyclically Strained on Physiological Stiffness

(A) Analysis of pillar displacement on different pillar dimensions in systole and diastole. High pillars (2.3 µm) are significantly pulled further in peak systole, while 2.3- and 1.7-µm-tall pillars are being pulled to the same displacement during diastole, indicating cyclic stretching of (mechanosensitive) intracellular elements (green circle). No change is observed between 0.8- and 1.3-µm-tall pillars, suggesting constitutive stretching of intracellular elements (red circle). Error bars: SD. N > 12 cells with >100 pillars analyzed per cell for all conditions.

(B) Comparison of NRC cell edge movements after thresholding (as indicator of contractile state) and RFP/GFP ratio of a talin-TS or control sensor (CS) at adhesions indicates cyclic loss of FRET (i.e., increase of tension) together with the contractions that are absent with the CS.

(C) Pearson coefficient was calculated for the normalized ratiometric signal (average of all adhesions) and the normalized cell edge position (see Figure S5C) for the tension sensor on different stiffness as well as the CS.

(D) Comparison of the RFP/GFP ratio during diastole and peak systole (see Figure S5C), averaged over all contractions during the movie. Boxplots: Tukey; N > 12 cells.

(E) Based on our data, we propose that cardiomyocytes sense the matrix rigidity using non-muscle and muscle myosin contractions, which work in series. On physiological stiffness, this will cause cyclic stretching of mechanosensitive proteins, such as talin. Increased stiffness, or enhanced non-muscle myosin activity, will result in a loss of cyclic mechanosignals. ECM, extracellular matrix.

*p < 0.05; **p < 0.01; ****p < 0.0001; ns, not significant; p values from ANOVA and Tukey correction for multiple comparisons. See also Figure S8.

Linke, 2008; Puchner et al., 2008; Sawada et al., 2006). Talin is an adaptor protein that directly links integrins with the actin cytoskeleton and stretching of talin by >100 nm has been demonstrated *in silico*, *in vitro*, and *in vivo* (Yao et al., 2016; Margadant et al., 2011). A talin molecular tension sensor (talin-TS) using the flagelliform elastic domain with a dynamic force range of <6 pN, which is insensitive to endogenous talin, indicated higher tension on stiff than on soft surfaces in a range of different cells (Kumar et al., 2016), although a subset of talin molecules experienced forces of >7 pN (Austen et al., 2015). Dynamic talin stretching opens cryptic binding sites for vinculin to allow adhesion reinforcement and mechanosensing (Kumar et al., 2016; Margadant et al., 2011; del Rio et al., 2009). Because our data indicated stronger enrichment of vinculin at stiff compared with soft pillars (Figure S6), we hypothesized that talin stretching could be a key factor in cardiomyocyte rigidity sensing.

The unfolding of the R3 domain, the initial mechanosensor domain harboring vinculin binding sites, occurs at forces below 5 pN (Yao et al., 2016). Therefore, we tested whether we could detect differences in talin tension in cardiomyocytes on substrates of different stiffnesses, using the talin-TS with the <6 pN elastic flagelliform domain (Kumar et al., 2016). Measuring this sensor in fixed cells using a confocal microscope with spectral detector to record simultaneously donor and acceptor fluorescence after donor excitation, we could indeed detect a decrease of FRET (i.e., an increase of tension) with increasing stiffness (Figures S8A and S8B). To analyze the tension on talin dynamically we next recorded movies of adhesions at 40 Hz and compared the cell edge movements to track the myofibrillar contractions with changes to FRET ratios at adhesions (Figures 4B–4D and S8C).

To quantify the differences, we calculated the Pearson correlation coefficient, as well as the ratio of red fluorescent protein (RFP)/GFP during diastole compared with the following systole (Figure S8C) and calculated the average between all contractions over the length of the movie (1,000 frames). On

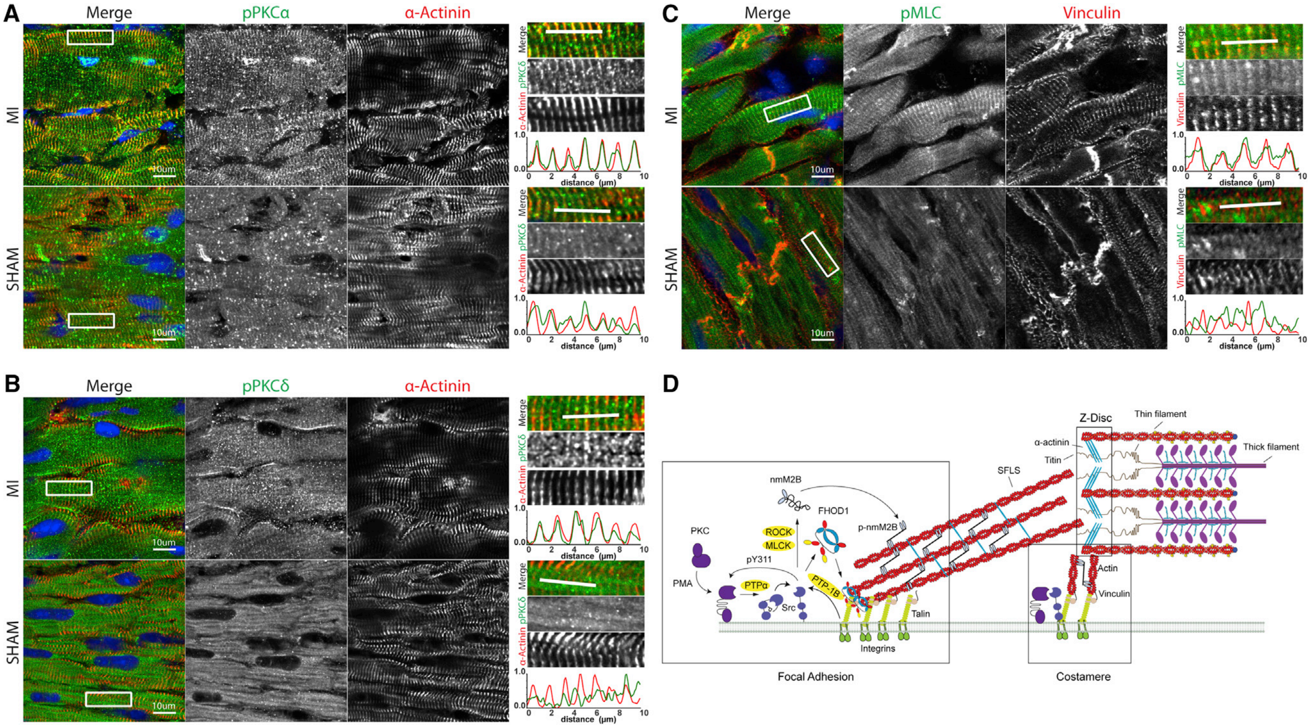

Figure 5. Elevated PKCδ and Non-muscle Myosin Activity after Myocardial Infarction and in Dilated Cardiomyopathy

Mouse hearts, 4 weeks after myocardial infarction (MI) or sham-operated mice (A–C) were stained with antibodies against PKCα, PKCδ, pMLC, α-actinin, or vinculin. White boxes indicate the regions of the close ups, shown on the right hand side of the figure panels. White lines indicate the regions for the line scan.

(A) PKCα co-localizes with α-actinin in sham as well as in MI samples.

(B) PKCδ activity is elevated at the Z disc in MI samples but only diffusely localized in sham-operated mice.

(C) Similarly, pMLC staining intensity is found in striations that partially overlap with vinculin or α-actinin (not shown) and likely localize between Z disc and costameres.

(D) Cartoon indicating the localization of the relevant molecules and activating pathways. Briefly, PKC activates Src, which again modulates PKC activity through tyrosine phosphorylation. Src activation in turn leads to alteration of non-myofibrillar tension through FHOD1 and non-muscle myosin. Src also associate with β1-integrin tails, resulting in its activation. Activating pathways, which are discussed in the main text and are not part of the current study but have been published previously, are indicated in yellow. Focal adhesions and stress-fiber-like structures as found in cultured cardiomyocytes are indicated on the left and costameres on the right. See also Figure S9.

intermediate stiffness (6 kPa), we detected comparably high correlation, as well as ~35% higher tension on the sensor in systole versus diastole. Both the tension ratio and correlation were lower on 20 kPa surfaces and there were no detectable differences between the control sensor and the tension sensor on 1 kPa or 130 kPa surfaces. Also, both measurements were reduced after treatment with BIS I or PP2 (Figures S8D and S8E), confirming that cyclical stretching of talin is regulated downstream of PKC and Src activity. Therefore, our data indicated that non-muscle and muscle myosin derived forces together result in cyclical stretching of talin during myofibrillar contractions on physiological stiffness but continuous extension on fibrotic stiffness (see model in Figure 4E).

PKCδ and Non-muscle Myosin Activity Are Enhanced in Cardiac Disease

Cardiac fibrosis is one of the key responses to heart diseases from MI to cardiomyopathies. It includes scar tissue formation in the infarcted region, perivascular and interstitial fibrosis in non-infarcted regions in cases of MI, perivascular and interstitial fibrosis in cardiomyopathies, as well as replacement or focal fibrosis in the late phase of remodeling leading to heart failure. While fibrosis helps to counteract rupture, aneurysm, or dilation, it also leads to a loss of elasticity of the heart and elastic moduli of >100 kPa have been reported for fibrotic tissue. Interestingly, elevated PKC levels have been reported for both MI (PKCα and δ) as well as dilated cardiomyopathy (PKCα) (Lange et al., 2016; Wang et al., 2003b; Simonis et al., 2002).

In support of this, we found PKCδ and PKCα in a striated pattern, partially overlapping with α-actinin, in infarcted mouse hearts (Figures 5A and 5B). This indicated localization to integrin adhesion sites at costameres in agreement with reported interactions of PKC and integrins (Chae et al., 2010; Disatnik and Rando, 1999; Haller et al., 1998). In the sham-operated controls this pattern was less pronounced for PKCα and absent in case of PKCδ. Similarly, phosphorylated non-muscle myosin light chain was present in infarcted hearts at a localization that was partially overlapping with α-actinin (not shown) and co-staining with vinculin confirmed strong costameric localization, which again was absent in the sham-operated mice (Figure 5C). While in the sham hearts, a faint striated pattern could be observed, the staining was not overlapping, but rather displayed double peaks in

between the vinculin striations (Figure 5C, line scans) and therefore was likely caused by cross-reactivity of the antibody with the cardiac myosin light chain at the A bands. A comparable result was also obtained when we examined left ventricles of the MLP knockout mouse, a widely studied model for dilated cardiomyopathy (Figure S9) (Arber et al., 1997). Non-muscle myosin was again prominent at the costameres and additionally also the intercalated disc but was only detected in a diffuse cytoplasmic staining or alternating with the α-actinin staining pattern. Therefore, we find that signaling pathways leading to the activation of rigidity sensing could also play a role in cardiac disease models where we detect enhanced PKC and non-muscle myosin activity at integrin adhesion sites. Together our results demonstrate a finely tuned system that is sensitive toward both increases in the stiffness of the environment as well as humoral factors that lead to elevated non-muscle myosin contractility.

DISCUSSION

Using a combination of nanopillars and flat surfaces with defined stiffness, we identify here a mechanism for cardiomyocyte rigidity sensing that relies on a combination of non-muscle myosin and muscle myosin contractions and can be activated by PKC signaling. PKC activates Src, which in a feedback mechanism modulates PKC activity through tyrosine phosphorylation. Src activation in turn leads to alteration of non-myofibrillar tension through FHOD1 and non-muscle myosin. This affects the tension on talin, thereby modulating the mechanical response by recruitment of vinculin to cryptic binding sites, which enhances the stability of the integrin-talin-actin link (del Rio et al., 2009). It is noteworthy that the effect on the stability of the talin-integrin interaction will likely feedback into Src activity, since Src is known to associate with β1-integrin tails, resulting in its activation, possibly through dephosphorylation by PTP-1B (Das et al., 2014) (see also cartoon in Figure 5D).

Importantly, our data reveal three discrete regimes of mechanotransduction, with either unstretched, cyclically stretched, or constitutively stretched mechanosensors. These regimes cover the stiffness of the fetal, adult, and fibrotic heart, respectively, and are regulated by the tension generated by non-muscle myosin (Huyer et al., 2015; Majkut et al., 2013; Jacot et al., 2010; Engler et al., 2008).

Previous work has highlighted the importance of myofibrillar tension (especially the mechanical relaxation) for cardiac health, whereby the integrated tension over the myofibrillar contraction can be used to predict whether sarcomeric mutations lead to concentric or eccentric hypertrophy (Davis et al., 2016). Here, we identify a role for non-muscle myosin activity, which leads to enhanced baseline tension in fibrotic hearts after MI or in dilated cardiomyopathy. The increased non-myofibrillar tension acts together with the increased passive elasticity of the fibrotic heart to shift oscillating to constitutive mechanosignaling in heart disease. Our results are in broad agreement with previous studies from primary and stem cell derived cardiomyocytes, reporting higher stresses with increasing stiffness (McCain et al., 2014; Hersch et al., 2013; Hazeltine et al., 2012; Rodriguez et al., 2011; Jacot et al., 2008). However, the discovery of the different types of mechanotransduction, enabled by the high resolution of the nanopillars, as well as the range of stiffness chosen for this study have important implications on our understanding of cardiac disease pathways. The non-muscle myosin forces are expected to further modify the tension integral, reported by Davis et al. (2016), but future work will need to analyze the mechanosignaling response in relevant eccentric or concentric disease models in detail.

Non-muscle myosin is present in cardiomyocytes in two isoforms (NM II-B and C), where it was found at the Z discs (NM II-B) and intercalated discs (NM II-B and C) (Ma and Adelstein, 2012; Takeda et al., 2000; Tullio et al., 1997). We also find localization of the active non-muscle myosin light chain at the intercalated discs of healthy heart sections, but the activity is enhanced in diseased hearts. Similarly, we find active non-muscle myosin at the costameres only in diseased hearts. Because this signal is partially overlapping with α-actinin, it is possible that the previously described Z-disc signal is in fact coming from the costameres. NM II-C is only present in small quantities and its genetic ablation has no obvious effects. Deletion of NM II-B, in contrast, is embryonic lethal at E14.5 and cardiac myocyte specific knockouts lead to progressive hypertrophic cardiomyopathy with disruption of the intercalated discs (Ma and Adelstein, 2012; Ma et al., 2009; Takeda et al., 2000; Tullio et al., 1997). This suggests a role for NM II-B for myofibrillar anchorage that is in agreement with the localization of active non-muscle myosin light chain at the cell edge and at force transduction sites that we find in isolated cardiomyocytes. However, our data further indicate that NM II-B-generated forces can be measured by mechanosensitive proteins and shift mechanotransduction regimes. This indicates that aberrant mechanosensing is a contributing factor to cardiac remodeling and, in the long term, to heart failure. Further, this suggests a future possibility of pharmacological targeting of non-myofibrillar force production in cardiac disease to correct dysfunctional mechanotransduction and reduce the risk of end-stage heart failure.

While our study focuses here on cardiomyocyte rigidity sensing, co-operation of different myosin isoforms and subtypes is evident in many cell types and tissues and these motor proteins differ in actin binding, enzymatic activity, as well as their regulation (Billington et al., 2013; Norstrom et al., 2010; Kovacs et al., 2007; Kim et al., 2005; Kovacs et al., 2003; Wang et al., 2003a). Moreover, de-regulation of myosin activities and/or expression has been associated with a range of diseases (Newell-Litwa et al., 2015). Therefore, it is likely that comparable multimodal mechanosensing mechanisms are in place in other cell types as well, albeit with different dynamics.

The combination of its intrinsic deformability, its role in signaling, and the positioning at the interface between integrins and actin put talin in a perfect position to measure subtle changes in mechanical properties (Yao et al., 2016; Das et al., 2014; del Rio et al., 2009). Here, the use of molecular tension sensors allowed us to establish the stiffness-dependent tension on talin with high temporal resolution. However, in addition to talin, other proteins have been demonstrated to be stretched by force and contribute to mechanical signaling as well, including p130Cas, focal adhesion kinase, and titin, especially the titin kinase domain and titin's I-band region, including the N2B, N2A, and PEVK domains, whereby, intriguingly, the stiffness of the latter can be adjusted through phosphorylation by PKCα (Zhou et al., 2015; Anderson et al., 2010; Linke, 2008; Puchner et al.,

2008; Sawada et al., 2006). Therefore, it is possible that other mechanosensitive proteins respond in a similar manner and indeed atomic force microscopy force extension traces for titin kinase and talin suggest a similar stiffness of the mechanosensitive domains (del Rio et al., 2009; Puchner et al., 2008). Importantly, unfolding of mechanosensors can mediate the binding and targeting of proteins that are not direct mechanosensors, such as the LIM domain protein PINCH1, which is part of the ILK-PINCH-Parvin complex at the costamere and the Z disc and involved in embryonic heart development (Li et al., 2012; Jani and Schock, 2009). Other cardiac LIM domain proteins are known to shuttle between the cytoplasm and the nucleus and act as transcriptional co-activators and therefore could potentially modify gene expression patterns in response to mechanical stimuli (Li et al., 2012; Schiller et al., 2011).

Together our data indicate a finely tuned system that is influenced by mechanical and chemical factors to regulate cardiomyocyte signaling through non-muscle myosin activity at integrin adhesion sites. Intriguingly, this also highlights the potential of reducing the impact of cardiac fibrosis by targeting non-myofibrillar force production in cardiac disease to modify aberrant mechanotransduction.

STAR★METHODS

Detailed methods are provided in the online version of this paper and include the following:

- KEY RESOURCES TABLE
- CONTACT FOR REAGENT AND RESOURCE SHARING
- EXPERIMENTAL MODEL AND SUBJECT DETAILS
  - Neonatal Rat Cardiomyocytes
  - Myocardial Infarction
- METHOD DETAILS
  - Cell Culture, Immunostaining and Microscopy
  - PDMS Substrates and Nanopillars
  - Rheology
  - Image Analysis
- QUANTIFICATION AND STATISTICAL ANALYSIS

SUPPLEMENTAL INFORMATION

Supplemental Information includes nine figures and four movies and can be found with this article online at https://doi.org/10.1016/j.devcel.2017.12.024.

ACKNOWLEDGMENTS

We would like to acknowledge the Nikon Imaging Centre at King's College London, as well as Brian Stramer and Fiona Kenny for access and help with microscopy. We thank Ay Lin Kho for help with cardiomyocyte isolations and Martin Schwartz for the talin tension sensor constructs. T.I. was supported by a British Heart Foundation Intermediate Basic Science Research Fellowship (FS/14/30/30917). E.E. was supported by the British Heart Foundation. M.Z. was partially supported by the British Heart Foundation (PG/12/26/29477, PG/17/39/33027). S.C. was supported by a Royal Society University Research Fellowship and Human Science Frontiers Program (RGP0035/2016).

AUTHOR CONTRIBUTIONS

Conceptualization, M.S. and T.I.; Methodology, P.P., J.G., S.C., J. Hone, E.E., M.Z., and T.I.; Investigation, P.P., W.H., J. Hu, N.A., L.H., S.C., W.V.M., and T.I.; Writing – Original Draft, Review & Editing, T.I.; Resources, S.C., E.E., M.Z., M.S., J. Hone, and T.I.; Supervision, S.C., M.Z., J.G., J. Hone, and T.I.

DECLARATION OF INTERESTS

The authors declare no competing financial interests.

Received: June 15, 2017
Revised: November 9, 2017
Accepted: December 22, 2017
Published: January 25, 2018

REFERENCES

Al Haj, A., Mazur, A.J., Radaszkiewicz, K., Radaszkiewicz, T., Makowiecka, A., Stopschinski, B.E., Schonichen, A., Geyer, M., and Mannherz, H.G. (2015). Distribution of formins in cardiac muscle: FHOD1 is a component of intercalated discs and costameres. Eur. J. Cell Biol. *94*, 101–113.

Anderson, B.R., Bogomolovas, J., Labeit, S., and Granzier, H. (2010). The effects of PKCalpha phosphorylation on the extensibility of titin's PEVK element. J. Struct. Biol. *170*, 270–277.

Arber, S., Hunter, J.J., Ross, J., Jr., Hongo, M., Sansig, G., Borg, J., Perriard, J.C., Chien, K.R., and Caroni, P. (1997). MLP-deficient mice exhibit a disruption of cardiac cytoarchitectural organization, dilated cardiomyopathy, and heart failure. Cell *88*, 393–403.

Austen, K., Ringer, P., Mehlich, A., Chrostek-Grashoff, A., Kluger, C., Klingner, C., Sabass, B., Zent, R., Rief, M., and Grashoff, C. (2015). Extracellular rigidity sensing by talin isoform-specific mechanical linkages. Nat. Cell Biol. *17*, 1597–1606.

Billington, N., Wang, A., Mao, J., Adelstein, R.S., and Sellers, J.R. (2013). Characterization of three full-length human nonmuscle myosin II paralogs. J. Biol. Chem. *288*, 33398–33410.

Birukov, K.G., Csortos, C., Marzilli, L., Dudek, S., Ma, S.F., Bresnick, A.R., Verin, A.D., Cotter, R.J., and Garcia, J.G. (2001). Differential regulation of alternatively spliced endothelial cell myosin light chain kinase isoforms by p60(Src). J. Biol. Chem. *276*, 8567–8573.

Bishop, J.E., and Laurent, G.J. (1995). Collagen turnover and its regulation in the normal and hypertrophying heart. Eur. Heart J. *16* (*Suppl C*), 38–44.

Brandt, D.T., Goerke, A., Heuer, M., Gimona, M., Leitges, M., Kremmer, E., Lammers, R., Haller, H., and Mischak, H. (2003). Protein kinase C delta induces Src kinase activity via activation of the protein tyrosine phosphatase PTP alpha. J. Biol. Chem. *278*, 34073–34078.

Braz, J.C., Bueno, O.F., De Windt, L.J., and Molkentin, J.D. (2002). PKC alpha regulates the hypertrophic growth of cardiomyocytes through extracellular signal-regulated kinase1/2 (ERK1/2). J. Cell Biol. *156*, 905–919.

Cai, D., Mulle, J.G., and Yue, D.T. (1997). Inhibition of recombinant Ca2+ channels by benzothiazepines and phenylalkylamines: class-specific pharmacology and underlying molecular determinants. Mol. Pharmacol. *51*, 872–881.

Chae, Y.C., Kim, K.L., Ha, S.H., Kim, J., Suh, P.G., and Ryu, S.H. (2010). Protein kinase Cdelta-mediated phosphorylation of phospholipase D controls integrin-mediated cell spreading. Mol. Cell. Biol. *30*, 5086–5098.

Chan, C.E., and Odde, D.J. (2008). Traction dynamics of filopodia on compliant substrates. Science *322*, 1687–1691.

Das, M., Ithychanda, S., Qin, J., and Plow, E.F. (2014). Mechanisms of talin-dependent integrin signaling and crosstalk. Biochim. Biophys. Acta *1838*, 579–588.

Davis, J., Davis, L.C., Correll, R.N., Makarewich, C.A., Schwanekamp, J.A., Moussavi-Harami, F., Wang, D., York, A.J., Wu, H., Houser, S.R., et al. (2016). A tension-based model distinguishes hypertrophic versus dilated cardiomyopathy. Cell *165*, 1147–1159.

del Rio, A., Perez-Jimenez, R., Liu, R., Roca-Cusachs, P., Fernandez, J.M., and Sheetz, M.P. (2009). Stretching single talin rod molecules activates vinculin binding. Science *323*, 638–641.

Disatnik, M.H., and Rando, T.A. (1999). Integrin-mediated muscle cell spreading. The role of protein kinase c in outside-in and inside-out signaling and evidence of integrin cross-talk. J. Biol. Chem. *274*, 32486–32492.

Dwyer, J., Pluess, M., Iskratsch, T., Dos Remedios, C.G., and Ehler, E. (2014). The formin FHOD1 in cardiomyocytes. Anat. Rec. (Hoboken) *297*, 1560–1570.

Elosegui-Artola, A., Bazellieres, E., Allen, M.D., Andreu, I., Oria, R., Sunyer, R., Gomm, J.J., Marshall, J.F., Jones, J.L., Trepat, X., et al. (2014). Rigidity sensing and adaptation through regulation of integrin types. Nat. Mater. *13*, 631–637.

Engler, A.J., Carag-Krieger, C., Johnson, C.P., Raab, M., Tang, H.Y., Speicher, D.W., Sanger, J.W., Sanger, J.M., and Discher, D.E. (2008). Embryonic cardiomyocytes beat best on a matrix with heart-like elasticity: scar-like rigidity inhibits beating. J. Cell Sci. *121*, 3794–3802.

Enyeart, J.J., Mlinar, B., and Enyeart, J.A. (1993). T-type Ca2+ channels are required for adrenocorticotropin-stimulated cortisol production by bovine adrenal zona fasciculata cells. Mol. Endocrinol. *7*, 1031–1040.

Fujita, H., Labeit, D., Gerull, B., Labeit, S., and Granzier, H.L. (2004). Titin isoform-dependent effect of calcium on passive myocardial tension. Am. J. Physiol. Heart Circ. Physiol. *287*, H2528–H2534.

Ghassemi, S., Meacci, G., Liu, S., Gondarenko, A.A., Mathur, A., Roca-Cusachs, P., Sheetz, M.P., and Hone, J. (2012). Cells test substrate rigidity by local contractions on submicrometer pillars. Proc. Natl. Acad. Sci. USA *109*, 5328–5333.

Ghibaudo, M., Saez, A., Trichet, L., Xayaphoummine, A., Browaeys, J., Silberzan, P., Buguin, A., and Ladoux, B. (2008). Traction forces and rigidity sensing regulate cell functions. Soft Matter *4*, 1836–1843.

Haller, H., Lindschau, C., Maasch, C., Olthoff, H., Kurscheid, D., and Luft, F.C. (1998). Integrin-induced protein kinase Calpha and Cepsilon translocation to focal adhesions mediates vascular smooth muscle cell spreading. Circ. Res. *82*, 157–165.

Hazeltine, L.B., Simmons, C.S., Salick, M.R., Lian, X., Badur, M.G., Han, W., Delgado, S.M., Wakatsuki, T., Crone, W.C., Pruitt, B.L., et al. (2012). Effects of substrate mechanics on contractility of cardiomyocytes generated from human pluripotent stem cells. Int. J. Cell Biol. *2012*, 508294.

Hersch, N., Wolters, B., Dreissen, G., Springer, R., Kirchgeßner, N., Merkel, R., and Hoffmann, B. (2013). The constant beat: cardiomyocytes adapt their forces by equal contraction upon environmental stiffening. Biol. Open *2*, 351–361.

Hill, A. (1938). The heat of shortening and the dynamic constants of muscle. Proc. R. Soc. London B Biol. Sci. *126*, 136–195.

Huyer, L.D., Montgomery, M., Zhao, Y., Xiao, Y., Conant, G., Korolj, A., and Radisic, M. (2015). Biomaterial based cardiac tissue engineering and its applications. Biomed. Mater. *10*, 034004.

Iskratsch, T., Reijntjes, S., Dwyer, J., Toselli, P., Dégano, I.R., Dominguez, I., and Ehler, E. (2013a). Two distinct phosphorylation events govern the function of muscle FHOD3. Cell. Mol. Life Sci. *70*, 893–908.

Iskratsch, T., Yu, C.H., Mathur, A., Liu, S., Stévenin, V., Dwyer, J., Hone, J., Ehler, E., and Sheetz, M. (2013b). FHOD1 is needed for directed forces and adhesion maturation during cell spreading and migration. Dev. Cell *27*, 545–559.

Iskratsch, T., Wolfenson, H., and Sheetz, M.P. (2014). Appreciating force and shape-the rise of mechanotransduction in cell biology. Nat. Rev. Mol. Cell Biol. *15*, 825–833.

Jacot, J.G., Martin, J.C., and Hunt, D.L. (2010). Mechanobiology of cardiomyocyte development. J. Biomech. *43*, 93–98.

Jacot, J.G., McCulloch, A.D., and Omens, J.H. (2008). Substrate stiffness affects the functional maturation of neonatal rat ventricular myocytes. Biophys. J. *95*, 3479–3487.

Jani, K., and Schock, F. (2009). Molecular mechanisms of mechanosensing in muscle development. Dev. Dyn. *238*, 1526–1534.

Keenan, C., and Kelleher, D. (1998). Protein kinase C and the cytoskeleton. Cell. Signal. *10*, 225–232.

Kim, K.Y., Kovacs, M., Kawamoto, S., Sellers, J.R., and Adelstein, R.S. (2005). Disease-associated mutations and alternative splicing alter the enzymatic and motile activity of nonmuscle myosins II-B and II-C. J. Biol. Chem. *280*, 22769–22775.

Kovacs, M., Thirumurugan, K., Knight, P.J., and Sellers, J.R. (2007). Load-dependent mechanism of nonmuscle myosin 2. Proc. Natl. Acad. Sci. USA *104*, 9994–9999.

Kovacs, M., Wang, F., Hu, A., Zhang, Y., and Sellers, J.R. (2003). Functional divergence of human cytoplasmic myosin II: kinetic characterization of the non-muscle IIA isoform. J. Biol. Chem. *278*, 38132–38140.

Kumar, A., Ouyang, M., Van den Dries, K., McGhee, E.J., Tanaka, K., Anderson, M.D., Groisman, A., Goult, B.T., Anderson, K.I., and Schwartz, M.A. (2016). Talin tension sensor reveals novel features of focal adhesion force transmission and mechanosensitivity. J. Cell Biol. *213*, 371–383.

Labeit, D., Watanabe, K., Witt, C., Fujita, H., Wu, Y., Lahmers, S., Funck, T., Labeit, S., and Granzier, H. (2003). Calcium-dependent molecular spring elements in the giant protein titin. Proc. Natl. Acad. Sci. USA *100*, 13716–13721.

Lahmers, S., Wu, Y., Call, D.R., Labeit, S., and Granzier, H. (2004). Developmental control of titin isoform expression and passive stiffness in fetal and neonatal myocardium. Circ. Res. *94*, 505–513.

Lange, S., Gehmlich, K., Lun, A.S., Blondelle, J., Hooper, C., Dalton, N.D., Alvarez, E.A., Zhang, X., Bang, M.L., Abassi, Y.A., et al. (2016). MLP and CARP are linked to chronic PKCalpha signalling in dilated cardiomyopathy. Nat. Commun. *7*, 12120.

Lee, H.H., Tien, S.C., Jou, T.S., Chang, Y.C., Jhong, J.G., and Chang, Z.F. (2010). Src-dependent phosphorylation of ROCK participates in regulation of focal adhesion dynamics. J. Cell Sci. *123*, 3368–3377.

Leuranguer, V., Monteil, A., Bourinet, E., Dayanithi, G., and Nargeot, J. (2000). T-type calcium currents in rat cardiomyocytes during postnatal development: contribution to hormone secretion. Am. J. Physiol. Heart Circ. Physiol. *279*, H2540–H2548.

Li, A., Ponten, F., and dos Remedios, C.G. (2012). The interactome of LIM domain proteins: the contributions of LIM domain proteins to heart failure and heart development. Proteomics *12*, 203–225.

Linke, W.A. (2008). Sense and stretchability: the role of titin and titin-associated proteins in myocardial stress-sensing and mechanical dysfunction. Cardiovasc. Res. *77*, 637–648.

Linke, W.A., Kulke, M., Li, H., Fujita-Becker, S., Neagoe, C., Manstein, D.J., Gautel, M., and Fernandez, J.M. (2002). PEVK domain of titin: an entropic spring with actin-binding properties. J. Struct. Biol. *137*, 194–205.

Lockhart, M., Wirrig, E., Phelps, A., and Wessels, A. (2011). Extracellular matrix and heart development. Birth Defects Res. A Clin. Mol. Teratol. *91*, 535–550.

Ma, X., and Adelstein, R.S. (2012). In vivo studies on nonmuscle myosin II expression and function in heart development. Front. Biosci. (Landmark Ed.) *17*, 545–555.

Ma, X., Takeda, K., Singh, A., Yu, Z.X., Zerfas, P., Blount, A., Liu, C., Towbin, J.A., Schneider, M.D., Adelstein, R.S., et al. (2009). Conditional ablation of nonmuscle myosin II-B delineates heart defects in adult mice. Circ. Res. *105*, 1102–1109.

Majkut, S., Idema, T., Swift, J., Krieger, C., Liu, A., and Discher, D.E. (2013). Heart-specific stiffening in early embryos parallels matrix and myosin expression to optimize beating. Curr. Biol. *23*, 2434–2439.

Margadant, F., Chew, L.L., Hu, X., Yu, H., Bate, N., Zhang, X., and Sheetz, M. (2011). Mechanotransduction in vivo by repeated talin stretch-relaxation events depends upon vinculin. PLoS Biol. *9*, e1001223.

McCain, M.L., Yuan, H., Pasqualini, F.S., Campbell, P.H., and Parker, K.K. (2014). Matrix elasticity regulates the optimal cardiac myocyte shape for contractility. Am. J. Physiol. Heart Circ. Physiol. *306*, H1525–H1539.

Meacci, G., Wolfenson, H., Liu, S., Stachowiak, M.R., Iskratsch, T., Mathur, A., Ghassemi, S., Gauthier, N., Tabdanov, E., Lohner, J., et al. (2016). alpha-Actinin links extracellular matrix rigidity-sensing contractile units with periodic cell-edge retractions. Mol. Biol. Cell *27*, 3471–3479.

Miranti, C.K., Ohno, S., and Brugge, J.S. (1999). Protein kinase C regulates integrin-induced activation of the extracellular regulated kinase pathway upstream of Shc. J. Biol. Chem. *274*, 10571–10581.

Munoz, J.P., Collao, A., Chiong, M., Maldonado, C., Adasme, T., Carrasco, L., Ocaranza, P., Bravo, R., Gonzalez, L., Diaz-Araya, G., et al. (2009). The transcription factor MEF2C mediates cardiomyocyte hypertrophy induced by IGF-1 signaling. Biochem. Biophys. Res. Commun. *388*, 155–160.

Nargeot, J. (2000). A tale of two (calcium) channels. Circ. Res. *86*, 613–615.

Newell-Litwa, K.A., Horwitz, R., and Lamers, M.L. (2015). Non-muscle myosin II in disease: mechanisms and therapeutic opportunities. Dis. Model. Mech. *8*, 1495–1515.

Norstrom, M.F., Smithback, P.A., and Rock, R.S. (2010). Unconventional processive mechanics of non-muscle myosin IIB. J. Biol. Chem. *285*, 26326–26334.

Puchner, E.M., Alexandrovich, A., Kho, A.L., Hensen, U., Schafer, L.V., Brandmeier, B., Grater, F., Grubmuller, H., Gaub, H.E., and Gautel, M. (2008). Mechanoenzymatics of titin kinase. Proc. Natl. Acad. Sci. USA *105*, 13385–13390.

Rezakhaniha, R., Agianniotis, A., Schrauwen, J.T., Griffa, A., Sage, D., Bouten, C.V., van de Vosse, F.N., Unser, M., and Stergiopulos, N. (2012). Experimental investigation of collagen waviness and orientation in the arterial adventitia using confocal laser scanning microscopy. Biomech. Model. Mechanobiol. *11*, 461–473.

Rienks, M., Papageorgiou, A.P., Frangogiannis, N.G., and Heymans, S. (2014). Myocardial extracellular matrix: an ever-changing and diverse entity. Circ. Res. *114*, 872–888.

Roca-Cusachs, P., Iskratsch, T., and Sheetz, M.P. (2012). Finding the weakest link: exploring integrin-mediated mechanical molecular pathways. J. Cell Sci. *125*, 3025–3038.

Rodriguez, A.G., Han, S.J., Regnier, M., and Sniadecki, N.J. (2011). Substrate stiffness increases twitch power of neonatal cardiomyocytes in correlation with changes in myofibril structure and intracellular calcium. Biophys. J. *101*, 2455–2464.

Rybin, V.O., Guo, J., Gertsberg, Z., Feinmark, S.J., and Steinberg, S.F. (2008). Phorbol 12-myristate 13-acetate-dependent protein kinase C delta-Tyr311 phosphorylation in cardiomyocyte caveolae. J. Biol. Chem. *283*, 17777–17788.

Sawada, Y., Tamada, M., Dubin-Thaler, B.J., Cherniavskaya, O., Sakai, R., Tanaka, S., and Sheetz, M.P. (2006). Force sensing by mechanical extension of the Src family kinase substrate p130Cas. Cell *127*, 1015–1026.

Schiller, H.B., Friedel, C.C., Boulegue, C., and Fassler, R. (2011). Quantitative proteomics of the integrin adhesome show a myosin II-dependent recruitment of LIM domain proteins. EMBO Rep. *12*, 259–266.

Schoen, I., Hu, W., Klotzsch, E., and Vogel, V. (2010). Probing cellular traction forces by micropillar arrays: contribution of substrate warping to pillar deflection. Nano Lett. *10*, 1823–1830.

Schultz Jel, J., Witt, S.A., Glascock, B.J., Nieman, M.L., Reiser, P.J., Nix, S.L., Kimball, T.R., and Doetschman, T. (2002). TGF-beta1 mediates the hypertrophic cardiomyocyte growth induced by angiotensin II. J. Clin. Invest. *109*, 787–796.

Shen, K., Ramirez, B., Mapes, B., Shen, G.R., Gokhale, V., Brown, M.E., Santarsiero, B., Ishii, Y., Dudek, S.M., Wang, T., et al. (2015). Structure-function analysis of the non-muscle myosin light chain kinase (nmMLCK) isoform by NMR spectroscopy and molecular modeling: influence of MYLK variants. PLoS One *10*, e0130515.

Simonis, G., Honold, J., Schwarz, K., Braun, M.U., and Strasser, R.H. (2002). Regulation of the isozymes of protein kinase C in the surviving rat myocardium after myocardial infarction: distinct modulation for PKC-alpha and for PKC-delta. Basic Res. Cardiol. *97*, 223–231.

Sipido, K.R., Carmeliet, E., and Van de Werf, F. (1998). T-type Ca2+ current as a trigger for Ca2+ release from the sarcoplasmic reticulum in guinea-pig ventricular myocytes. J. Physiol. *508* (Pt 2), 439–451.

Sirker, A., Murdoch, C.E., Protti, A., Sawyer, G.J., Santos, C.X., Martin, D., Zhang, X., Brewer, A.C., Zhang, M., and Shah, A.M. (2016). Cell-specific effects of Nox2 on the acute and chronic response to myocardial infarction. J. Mol. Cell. Cardiol. *98*, 11–17.

Soufen, H., Salemi, V., Aneas, I., Ramires, F., Benício, A., Benvenuti, L., Krieger, J., and Mady, C. (2008). Collagen content, but not the ratios of collagen type III/I mRNAs, differs among hypertensive, alcoholic, and idiopathic dilated cardiomyopathy. Braz. J. Med. Biol. Res. *41*, 1098–1104.

Szibor, M., Poling, J., Warnecke, H., Kubin, T., and Braun, T. (2014). Remodeling and dedifferentiation of adult cardiomyocytes during disease and regeneration. Cell. Mol. Life Sci. *71*, 1907–1916.

Tabdanov, E., Gondarenko, S., Kumari, S., Liapis, A., Dustin, M.L., Sheetz, M.P., Kam, L.C., and Iskratsch, T. (2015). Micropatterning of TCR and LFA-1 ligands reveals complementary effects on cytoskeleton mechanics in T cells. Integr. Biol. (Camb) *7*, 1272–1284.

Takeda, K., Yu, Z.X., Qian, S., Chin, T.K., Adelstein, R.S., and Ferrans, V.J. (2000). Nonmuscle myosin II localizes to the Z-lines and intercalated discs of cardiac muscle and to the Z-lines of skeletal muscle. Cell Motil. Cytoskeleton *46*, 59–68.

Taylor, J.M., Rovin, J.D., and Parsons, J.T. (2000). A role for focal adhesion kinase in phenylephrine-induced hypertrophy of rat ventricular cardiomyocytes. J. Biol. Chem. *275*, 19250–19257.

Tullio, A.N., Accili, D., Ferrans, V.J., Yu, Z.X., Takeda, K., Grinberg, A., Westphal, H., Preston, Y.A., and Adelstein, R.S. (1997). Nonmuscle myosin II-B is required for normal development of the mouse heart. Proc. Natl. Acad. Sci. USA *94*, 12407–12412.

Vijayan, K., Szotek, E.L., Martin, J.L., and Samarel, A.M. (2004). Protein kinase C-alpha-induced hypertrophy of neonatal rat ventricular myocytes. Am. J. Physiol. Heart Circ. Physiol. *287*, H2777–H2789.

Wang, F., Kovacs, M., Hu, A., Limouze, J., Harvey, E.V., and Sellers, J.R. (2003a). Kinetic mechanism of non-muscle myosin IIB: functional adaptations for tension generation and maintenance. J. Biol. Chem. *278*, 27439–27448.

Wang, J., Liu, X., Sentex, E., Takeda, N., and Dhalla, N.S. (2003b). Increased expression of protein kinase C isoforms in heart failure due to myocardial infarction. Am. J. Physiol. Heart Circ. Physiol. *284*, H2277–H2287.

Watkins, S.J., Borthwick, G.M., Oakenfull, R., Robson, A., and Arthur, H.M. (2012). Angiotensin II-induced cardiomyocyte hypertrophy in vitro is TAK1-dependent and Smad2/3-independent. Hypertens. Res. *35*, 393–398.

Wei, K., Serpooshan, V., Hurtado, C., Diez-Cunado, M., Zhao, M., Maruyama, S., Zhu, W., Fajardo, G., Noseda, M., Nakamura, K., et al. (2015). Epicardial FSTL1 reconstitution regenerates the adult mammalian heart. Nature *525*, 479–485.

Wolfenson, H., Meacci, G., Liu, S., Stachowiak, M.R., Iskratsch, T., Ghassemi, S., Roca-Cusachs, P., O'Shaughnessy, B., Hone, J., and Sheetz, M.P. (2016). Tropomyosin controls sarcomere-like contractions for rigidity sensing and suppressing growth on soft matrices. Nat. Cell Biol. *18*, 33–42.

Yao, M., Goult, B.T., Klapholz, B., Hu, X., Toseland, C.P., Guo, Y., Cong, P., Sheetz, M.P., and Yan, J. (2016). The mechanical response of talin. Nat. Commun. *7*, 11966.

Zhou, J., Aponte-Santamaria, C., Sturm, S., Bullerjahn, J.T., Bronowska, A., and Grater, F. (2015). Mechanism of focal adhesion kinase mechanosensing. PLoS Comput. Biol. *11*, e1004593.

STAR★METHODS

KEY RESOURCES TABLE

REAGENT or RESOURCE	SOURCE	IDENTIFIER
Antibodies		
anti-α-actinin	Sigma	Cat#A7811; RRID: AB_476766;
anti-Vinculin	Sigma	Cat# V9131; RRID: AB_477629;
anti-PKCα	BD Biosciences	Cat# 610108; RRID: AB_397514;
anti-non-muscle myosin IIB	Developmental Studies Hybridoma Bank	Cat# CMII 23; RRID: AB_528359;
anti-phospho-Src Family (Tyr416, pSFK)	Cell Signaling Technology	Cat# 2101; RRID: AB_331697;
anti-PKCα/β II (Thr638/641)	Cell Signaling Technology	Cat# 9375; RRID: AB_2284224;
anti-phospho-PKCδ/θ (Ser643/676)	Cell Signaling Technology	Cat# 9376; RRID: AB_2168834;
anti-phospho-PKCdelta (Tyr311)	Cell Signaling Technology	Cat# 2055; RRID: AB_330876;
anti-phospho-PKC (pan) (βII Ser660)	Cell Signaling Technology	Cat# 9371; RRID: AB_2168219;
anti-phospho-Myosin Light Chain (pMLC) T18/S19	Cell Signaling Technology	Cat# 3674; RRID: AB_2147464;
anti-phospho-Myosin Light Chain (pMLC) S19	Cell Signaling Technology	Cat# 3671; RRID: AB_330248;
anti-GAPDH	Abcam	
anti-pFHOD1	ECM Biosciences	Cat# FP3481; RRID: AB_2104507;
Chemicals, Peptides, and Recombinant Proteins		
Phenylephrine (PE, 100 μM)	Sigma	Cat# P1240000
Angiotensin II, (AT II, 200 nM)	Sigma	Cat# A9525
Phorbol 12-myristate 13-acetate (PMA, 200 nM)	Cayman Chemical	Cat# 10008014
Insulin-like growth factor 1 (IGF-1, 100 ng/ml),	Life Technologies	Cat# PHG0071
Transforming growth factor beta 1 (TGF-β1, 5 ng/ml)	PeproTech	Cat# AF-100-21C
Bisindolylmaleimide I (BIS I, used at 500 nM)	Sigma	Cat# B6292
Bis II (5 μM)	Sigma	Cat# B3056
PP2 (10 μM)	Cayman Chemical	Cat# 13198
(±)-Blebbistatin (50 μM)	Calbiochem	Cat# 203390
Verapamil (10 μM)	SCBT	Cat# sc-3590A

CONTACT FOR REAGENT AND RESOURCE SHARING

Further information and requests for resources and reagents should be directed to and will be fulfilled by the Lead Contact, Thomas Iskratsch (t.iskratsch@qmul.ac.uk).

EXPERIMENTAL MODEL AND SUBJECT DETAILS

Neonatal Rat Cardiomyocytes

Neonatal rat cardiomyocytes were prepared by a sequential digest method. Newborn rat hearts were dissected into ice-cold ADS buffer (116mM NaCl, 20mM Hepes, 0.8mM NaH_2PO_4, 5.6mM Glucose, 5.4 mM KCL, 0.8 mM $MgSO_4$). After hearts settled down to the bottom, hearts were washed once with ADS buffer. ADS buffer was then removed and hearts were incubated with 5ml enzyme solution in ADS (ES, 246U Collagenase and 0.6 mg Pancreatin / ml), for 5 min, at 37C under vigorous shaking. Supernatant was discarded. This step was followed by 5-6 digests, until hearts were completely digested. Each time 5ml fresh ES was added to the hearts and incubated 15min at 37C, under shaking. Hearts were pipetted up and down 30 times using a pasteur pipette. After settling down, supernatant was transferred into plating medium (65% DMEM, 17% M199, 10% Horse Serum, 5% FCS, 2% Glutamax, 1% Penecillin/Streptamycin (P/S)). Two digests each were combined in one tube with 20ml plating medium, then cleared through a 100μm cell strainer and spun down at 1200rpm for 5min at RT, before resuspended in 10ml plating medium. Cells were pooled together and pre-plated for 90min to enrich the cardiomyocytes. Cardiomyocytes were then plated onto the respective substrates as indicated in the text or figures. Medium was changed the next day to maintenance medium (77% DMEM, 18% M199, 2% Horse Serum, 2% Glutamax, 1% P/S), or serum starvation medium (as above, but excluding the Horse Serum).

Myocardial Infarction

C57BL6/J mice aged 10-12 weeks were subjected to permanent ligation of the left anterior descending coronary artery (LAD) for 4 weeks. "Sham" groups underwent similar surgery apart from LAD ligation (Sirker et al., 2016). Only female mice were used, because mortality is higher in male compared to female. All procedures were performed in accordance with the Guidance on the Operation of the Animals (Scientific Procedures) Act, 1986 (UK Home Office). Frozen sections (6 μm) from the non-infarcted LV segment were stained with the indicated antibodies.

METHOD DETAILS

Cell Culture, Immunostaining and Microscopy

For immunostaining, cells were fixed with 4% PFA for 10 minutes, permeabilized with 0.2% triton X-100 in PBS for 5 minutes, blocked with 5% BSA in PBS for 1h and stained in the antibody solutions in immunostaining buffer (20 mM Tris, 155 mM NaCl, 2 mM EGTA, 2 mM MgCl2, 1% BSA at pH 7.4) (Iskratsch et al., 2013a). Cells were washed three times for 10 minutes with PBS after each step and mounted in MOWIOL 4-88 (0.1g/ml in Glycerol/Water/Tris (0.2M, pH8.0) at a ratio of 1/1/2) containing a final concentration of 4% n-propyl gallate. Live cell imaging and imaging of multiwell plates was performed on an inverted Nikon Eclipse Ti-E microscope with a Nikon DS-Qi2 sCMOS camera and equipped with a Solent Scientific chamber with temperature and CO2 control. Confocal microscopy was performed on a Nikon A1R+ inverted microscope with GaAsP Detectors. Imaging of FRET sensors, as well as DIC imaging of cardiomyocytes, used for cell area measurements after PMA treatment, was performed on an inverted ZEISS LSM 880 microscope with temperature and CO2 control. Imaging of contraction dynamics on PDMS coated coverslips was performed on a Zeiss Axio Observer.Z1 microscope with dish heater, 100X NA1.4 oil objective, and Andor iXon Ultra camera.

PDMS Substrates and Nanopillars

PDMS pillar (500 nm diameter, 0.8, 1.3, 1.7 or 2.3 μm height, 1μm centre-to-centre) substrates were prepared by soft lithography from silicon masters. For fluorescent labelled pillars, CdSeS/ZnS alloyed quantum dots (490nm, Sigma) were spun first on the master 30s at 10,000rpm with a 150i spin processor (SPS), before the addition of PDMS. PDMS (Sylgard 184, Dow Corning) was mixed thoroughly with its curing agent (10:1), degassed, poured over the silicon master, placed upside-down on a plasma-treated coverslip-dish (Mattek), or coverslip 4-well dishes (Ibidi) and cured at 80C for 2 h. The mould was then removed and the pillars were incubated with fibronectin for 1h at 37C.

The Stiffness of the pillars was calculated as described by Ghibaudo et al but taking into account substrate warping as described by Schoen et al (Equations 1, 2, 3, 4, and 5) (Schoen et al., 2010; Ghibaudo et al., 2008).

$$k_{bend} = \frac{3}{64}\pi E \frac{D^4}{H^3}; \quad \text{(Equation 1)}$$

$$corr = \frac{\frac{16}{3}\left(\frac{L}{D}\right)^3}{\left(\frac{16}{3}\left(\frac{L}{D}\right)^3 + \frac{7+6\nu}{3}\frac{L}{D} + 8T_{tilt}(\nu)\left(\frac{L}{D}\right)^2\right)}; \quad \text{(Equation 2)}$$

$$T_{tilt}(\nu) = a\frac{(1+\nu)}{2\pi}2(1-\nu) + \left(1 - \frac{1}{4(1-\nu)}\right); \quad \text{(Equation 3)}$$

$$k = k_{bend} * corr; \quad \text{(Equation 4)}$$

$$E_{Eff} = \frac{9k}{4\pi a}; \quad \text{(Equation 5)}$$

Flat PDMS substrates were prepared by spin-coating Sylgard 184, Sylard 527 or mixtures at the Ratios of 1:5, 1:10 and 1:20 with a 150i spin processor (SPS), onto coverslips for western blotting samples, or microscope slides for placing into multiwell plates (Grace Biolabs). Before spin coating, Sylgard 527 was pre-cured at 70C for 30 minutes with intermitted mixing to achieve a comparable viscosity to the Sylgard 184 mixture.

Rheology

Oscillatory rheology was used to characterise the mechanics of the series of PDMS samples using a TA Discovery HR-3 hybrid rheometer. The samples were cured *in situ* at 80°C for 2hrs and the gelation was monitored by an oscillating time sweep, with

an oscillating frequency of 1Hz and an oscillating displacement of 1e-4rads. Once cured the samples were then kept at room temperature for 5 minutes to cool and were then characterised using an oscillating frequency and stress sweep, and by transient stress relaxation. The frequency sweeps were performed from 0.1-100Hz at an oscillating displacement of 1e-4rads and the stress sweeps were performed from 0.1-100Pa at a frequency of 1Hz. Finally stress relaxation was performed using a shear strain of 2% and a hold time of 300s.

Image Analysis

Pillar displacements were analysed with imageJ, using the NanoTracking plugin. An image of the pillars after removal of the cells with 10x trypsin was taken as reference for the non-displaced pillars. For contraction analysis, pillar displacements from spontaneous contracting cardiomyocytes were measured for the whole movie, using Matlab. From the data, the maximum displacement (systole) was compared to the subsequent minimum and to the non-displaced pillars. Noise levels were measured from pillars outside the cell and were 20.6±2.6, 16.9±4.5, 26.5±4.9 and 29.8±8.5nm for 0.8, 1.3, 1.7 and 2.3μm pillars, respectively. Therefore, all pillars that were displaced above 30nm during the movie were taken into account for the analysis. Statistics were calculated between cells. A comparable result was obtained when combining all pillar displacements.

For comparison of immunostaining with pillar displacements, a perfect grid was assumed and deviations from the grid were calculated from pillars outside the cell. For the measurement of line profiles, pillar displacements above the 90th percentile were identified and a mask created. The centre of the cell was marked manually and the line profiles were calculated along a line from the respective point to the centre.

For analysis of the tension sensor, the linear unmixed channels were processed with a Gaussian blur filter with a radius of 2px, using ImageJ. A RGB movie was created and registered to align adhesions over the contraction cycle. After registration, channels were separated and the ratio movie was generated by dividing the RFP by the GFP channel. ROIs were drawn over adhesion areas and the ratio pixel intensity was measured over the length of the movie. To measure cell edge movements and identify the timing of systole and diastole, a threshold movie was created from the GFP channel and a ROI at the cell edge was drawn and analysed using the particle tracker function. Ratio and cell edge data traces were detrended and normalized in Matlab, before calculation of Pearson correlation coefficient. For comparison of RFP/GFP ratio at systole/diastole, maximum and subsequent minimum peaks were identified from the cell edge trace and ratio at the minimum (diastole) was divided by the ratio at the maximum (systole). The data from all peaks over the length of the movie (except first and last, to avoid artefacts from incomplete contractions) was averaged and statistics were calculated between movies.

Cell area, cell morphology and staining intensity were analysed with cell profiler and a grey scale coded output mask was created, which was used to crop the images of the individual cells and delete the surroundings. Actin and α-actinin images of single cells were then analysed for filament alignment using the OrientationJ plugin for ImageJ (Rezakhaniha et al., 2012).

For analysis of sarcomeric shortening and velocity, kymographs were drawn over multiple sarcomeres from the cell edge inwards. Positions of the Z-discs were located for each timepoint by identifying peaks in the kymograph after Gaussian blurring. Z-disc positions over time were filtered with a Butterworth filter, before the differences between two Z-discs and the respective shortening velocity were calculated.

QUANTIFICATION AND STATISTICAL ANALYSIS

Data sets were tested for normal distribution using the Shapiro–Wilk test. All box plots are displayed as median (central line), upper and lower quartile (box), +/- 1.5 x inter quartile range (whiskers). All n-numbers and statistical tests are indicated in the figure legends. All statistic tests were performed with Graphpad Prism.

Developmental Cell, Volume *44*

Supplemental Information

Cardiomyocytes Sense Matrix Rigidity through a Combination of Muscle and Non-muscle Myosin Contractions

Pragati Pandey, William Hawkes, Junquiang Hu, William Valentine Megone, Julien Gautrot, Narayana Anilkumar, Min Zhang, Liisa Hirvonen, Susan Cox, Elisabeth Ehler, James Hone, Michael Sheetz, and Thomas Iskratsch

Supplementary Information

Figure S1

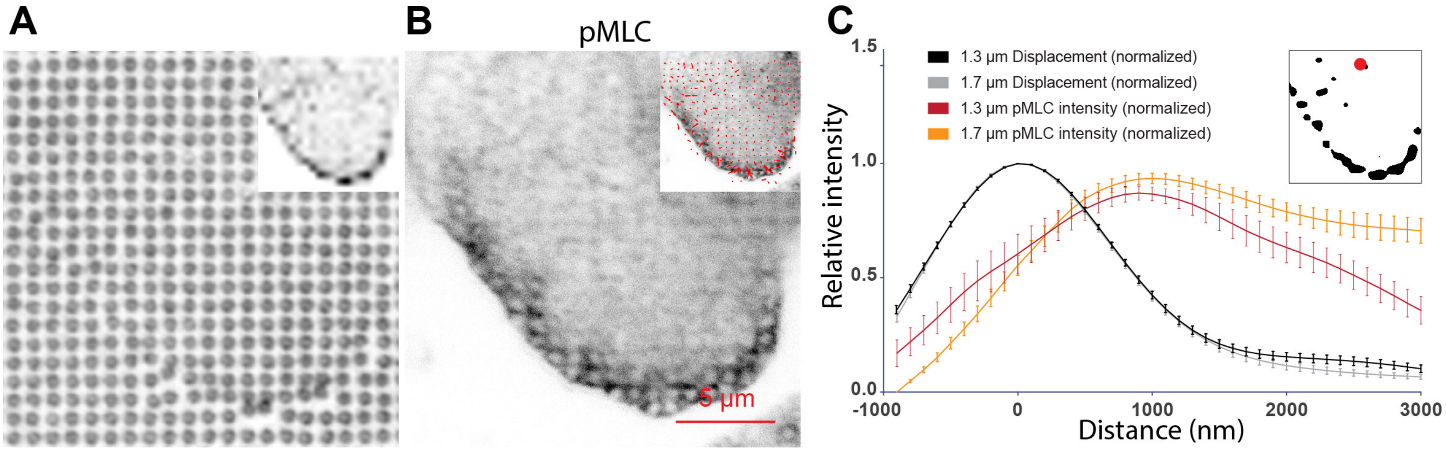

Figure S1: Non-muscle myosin light chain is active close to force transduction sites. Freshly isolated NRC were cultured on quantum dot labelled PDMS pillars (A, inset shows the grey scale coded displacement map) for 48 hours, fixed and stained for pMLC T18/S19 (B, overlaid with displacement vectors in inset). C) Radial profiles were calculated from displacements that were higher than the 90th percentile (inset, black areas) to the centre of the cell (inset, marked with red dot). Error bars: SEM; N=12 cells. Related to Figure 1&3.

Figure S2

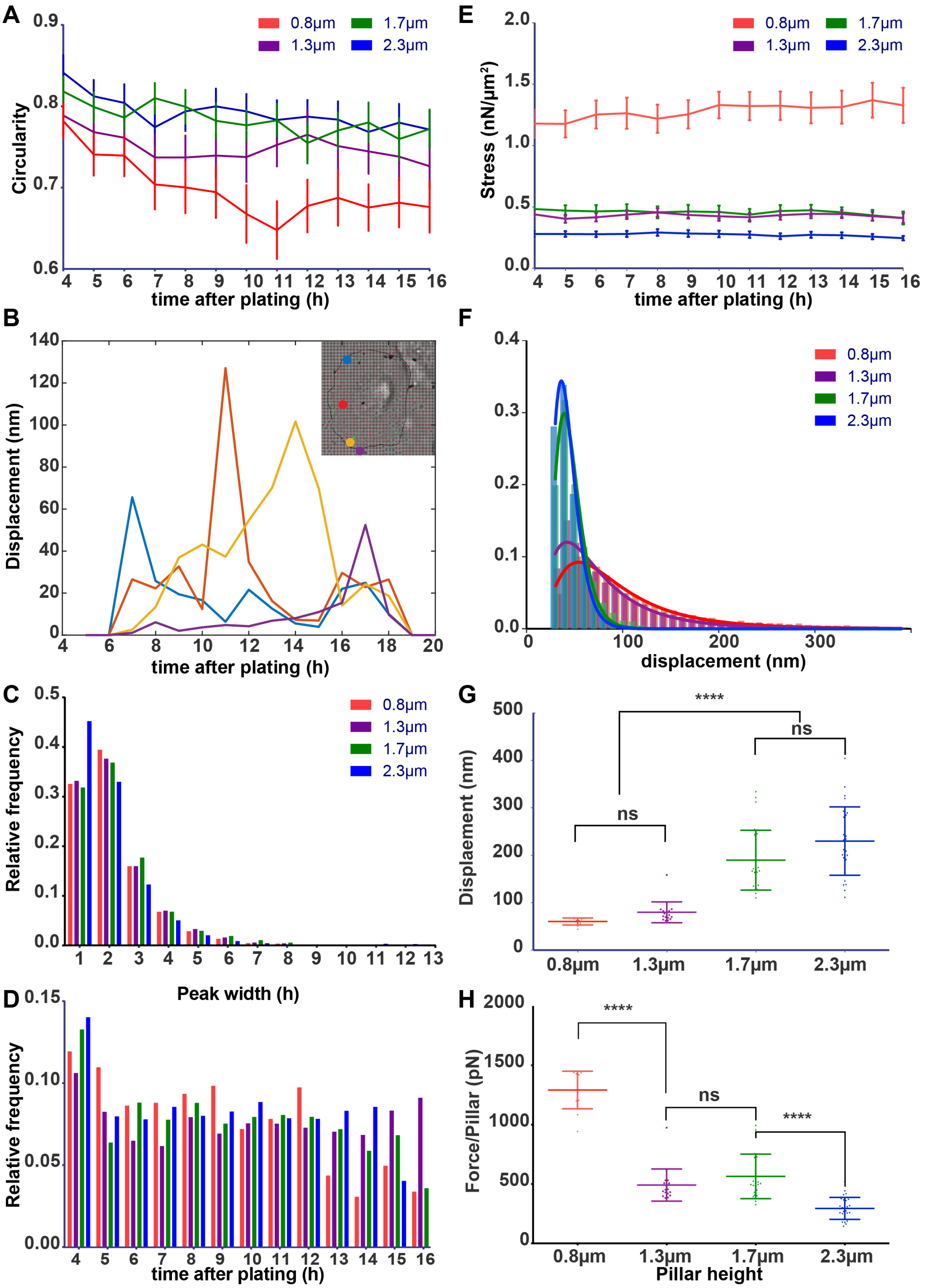

Figure S2 Analysis of spreading on Pillars of different stiffness. A) A reduction in circularity is observed during spreading, but faster and more pronounced on stiff pillars. B) Examples of individual pillar displacement traces for pillars indicated in the inset. C) Length of displacement events (i.e. peak width) shows no difference between pillar dimensions. D) Time of occurrence of the displacement peaks suggests that there is continued probing of the environment on all pillar dimensions. E) Because the cell is only applying the tension at the cell edge, there is no change of the stress over time. F) Pillar displacements follow a log-normal distribution. G-H) As expected for a log-normal distribution, the sharp change in displacement at equal force between the 1.3 µm and 1.7 µm high pillars is also detected when analysing the 90th percentile for each cell. Error bars: SEM (A,E) or mean ± SD (G,H); * $p<0.05$; ** $p<0.01$; *** $p<0.001$; **** $p<0.0001$; ns: not significant; P values from ANOVA and Tukey correction for multiple comparisons. Related to Figure 1.

Figure S3

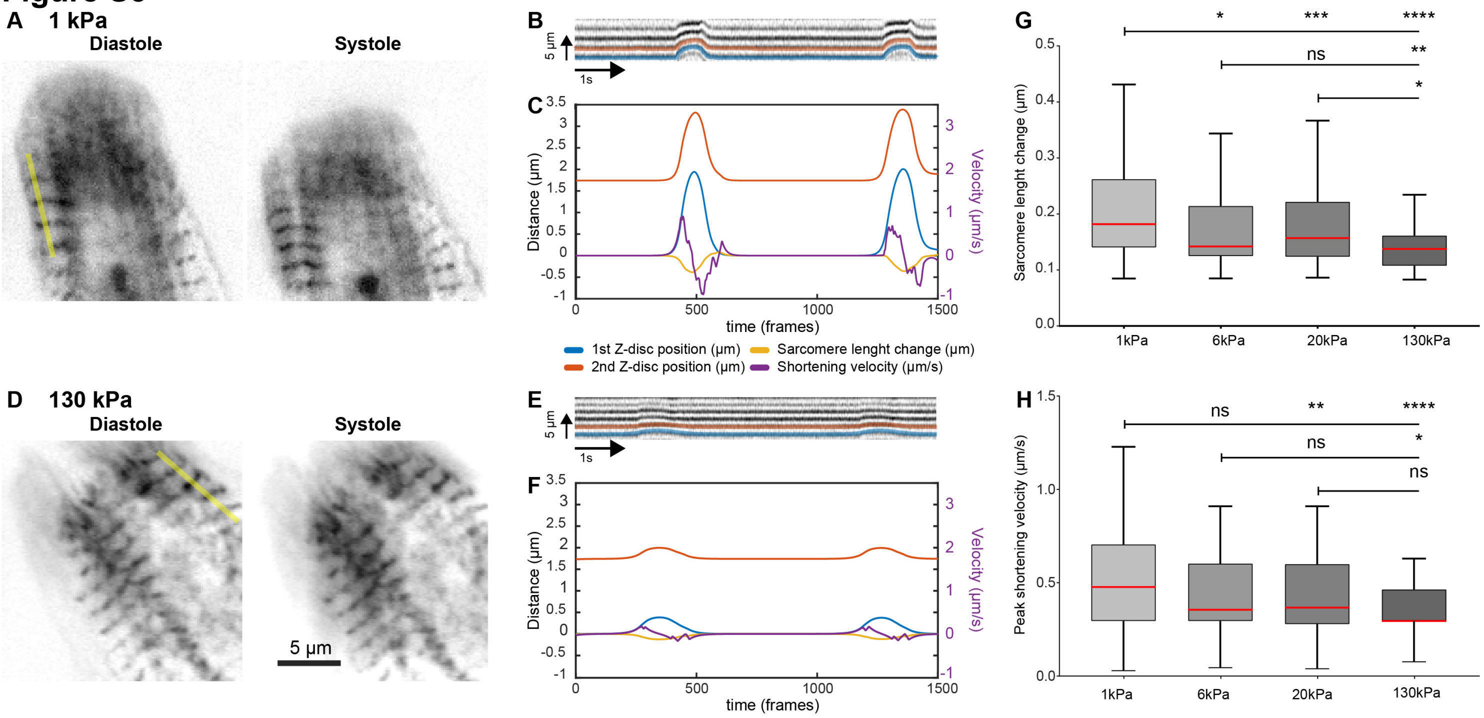

Figure S3 Contraction velocity is higher on soft PDMS. A) High speed movies (209 fps) of contracting, α-actinin GFP adenovirus infected neonatal cardiomyocytes were taken on PDMS surfaces with 1, 6, 20 and 130 kPa stiffness. A,D) Snapshots from cells during diastole and systole on 1 and 130 kPa respectively. B,E) Kymographs were drawn and used to measure z-disc positions during the contraction cycle and calculate sarcomeric length change and shortening velocity(C,F; A-C: 1kPa; D-E: 130kPa). G) Sarcomeres shorten to a larger extent on soft surfaces. H) Peak shortening velocity is higher on soft surfaces. n=11, 12, 11 and 13 cells for 1, 6, 20 and 130 kPa respectively. The first 4 sarcomeres from the cell edge were shortening to equal extent (data not shown) and hence were combined for the statistics in G and H.
* p<0.05; ** p<0.01; *** p<0.001; **** p<0.0001; ns: not significant; P values from ANOVA and Tukey correction for multiple comparisons. Related to Figure 1.

Figure S4

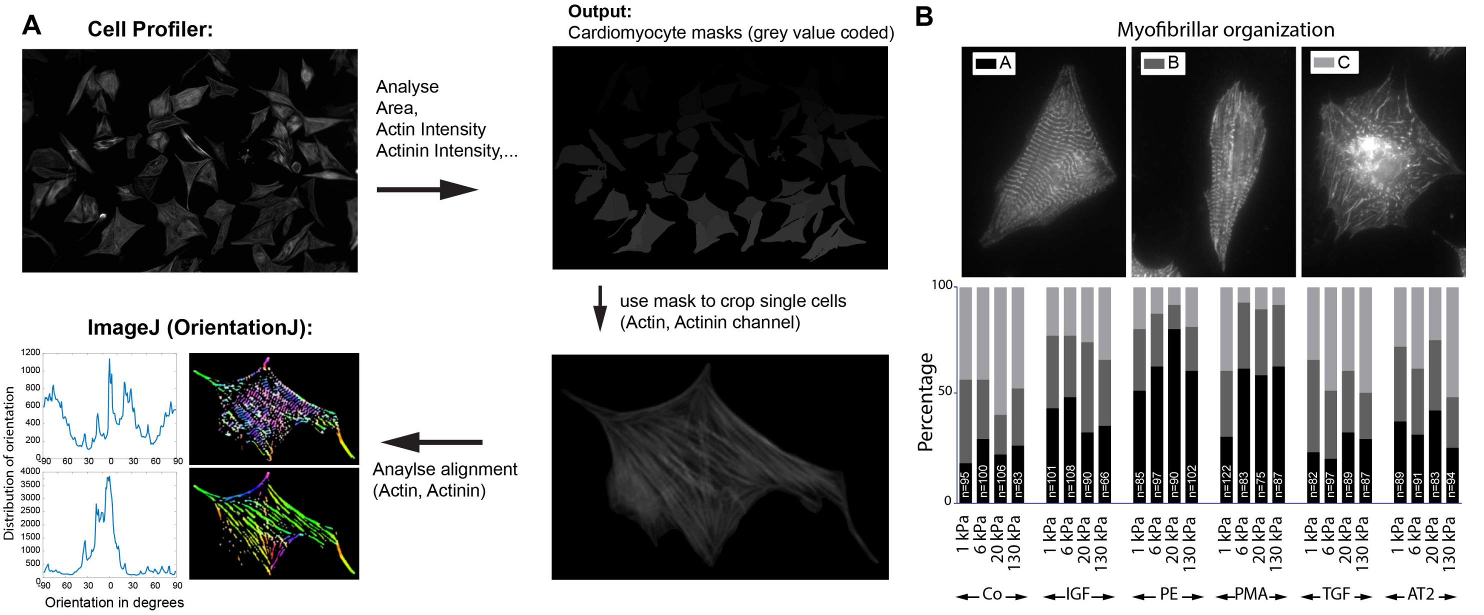

Figure S4 Analysis pipeline for multiwell assays. A) Images of NRC stained with α-actinin, Phalloidin and DAPI were taken on a large format CMOS camera (4908*3264px). Dapi and Phalloidin staining were used to segment cells in cell profiler and cardiomyocytes were selected based on α-actinin staining intensity. Cells were analysed for cell area, shape and staining intensities and a grey scale mask image was created as output image, based on which single cells were cropped. Images of single cells were then analysed with the orientationJ plugin in imageJ. Colours in the output images indicate orientation and saturation the coherency. Myofibrillar maturation results in higher anisotropy and fewer orientation peaks, with higher coherency values. The maximum value was aligned to 0 degrees, and averaged for all cells as indicator of the level of alignment. The result was confirmed manually by categorizing cells into three categories as displayed above (A: Mature myofibrils; B: mix of mature myofibrils with stress fibre-like structures (SFLS); C: mostly SFLS). N-numbers as indicated in the graph. Related to Figure 2.

Figure S5

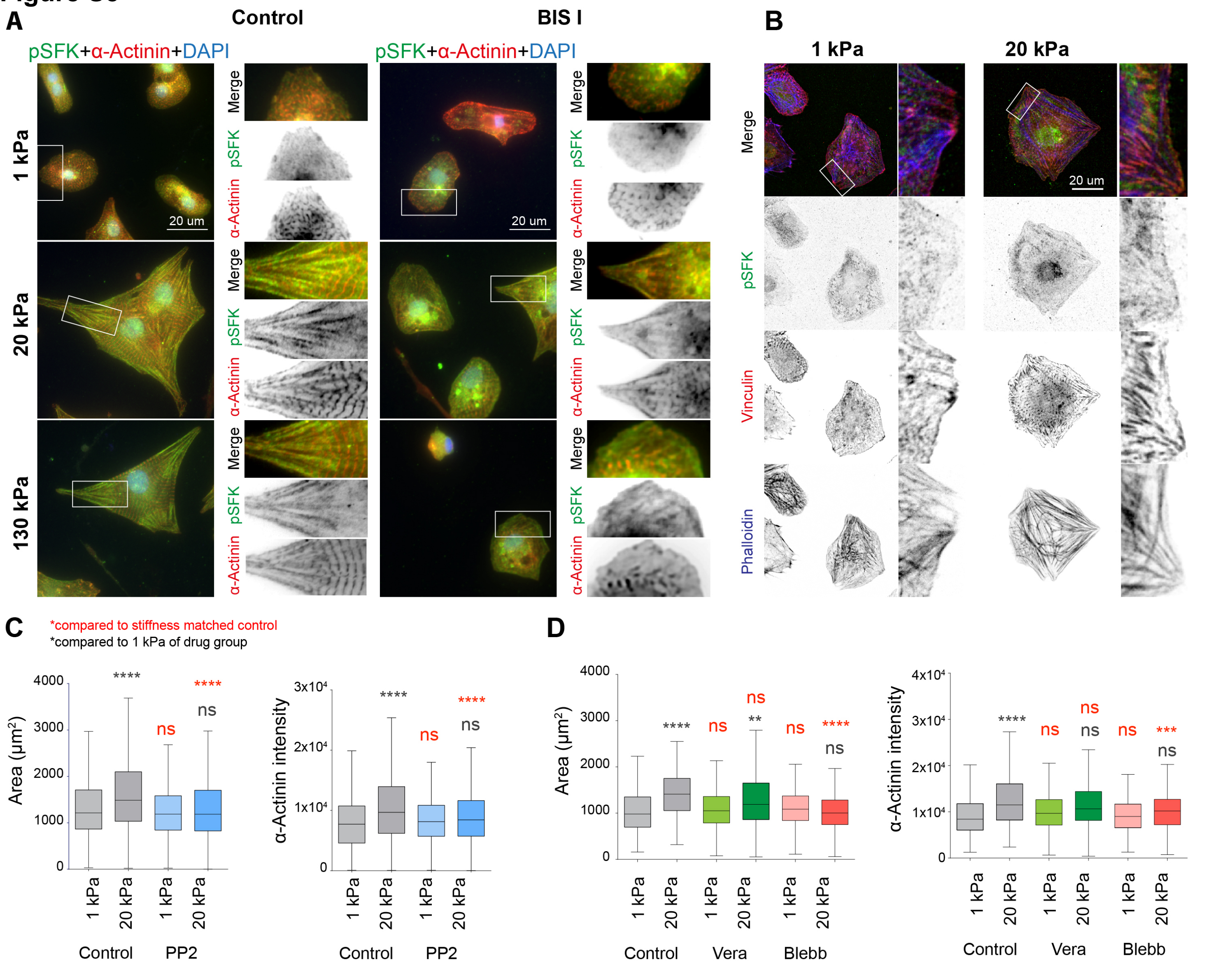

Figure S5: Src is activated downstream of PKC and required for cardiomyocyte rigidity sensing A) NRCs cultured on PDMS coverslips were stained for pSFK and α-actinin. Treatment with BIS I reduces the activity of active Src at the cell edge. B) During cardiomyocyte spreading the pSFK staining partially overlaps with Vinculin on stiff, but not on soft surfaces. C) Treatment with PP2 reduces the rigidity dependent differences in cell area and α-actinin staining intensity. D) The rigidity dependence of cell area and α-actinin staining intensity is reduced slightly after verapamil treatment (Vera) and abolished after blebbistatin treatment (Blebb). Error bars: SEM; N>100 cells for all conditions; * p<0.05; ** p<0.01; *** p<0.001; **** p<0.0001; ns: not significant; P values from ANOVA and Tukey correction for multiple comparisons. Related to Figure 3.

Figure S6

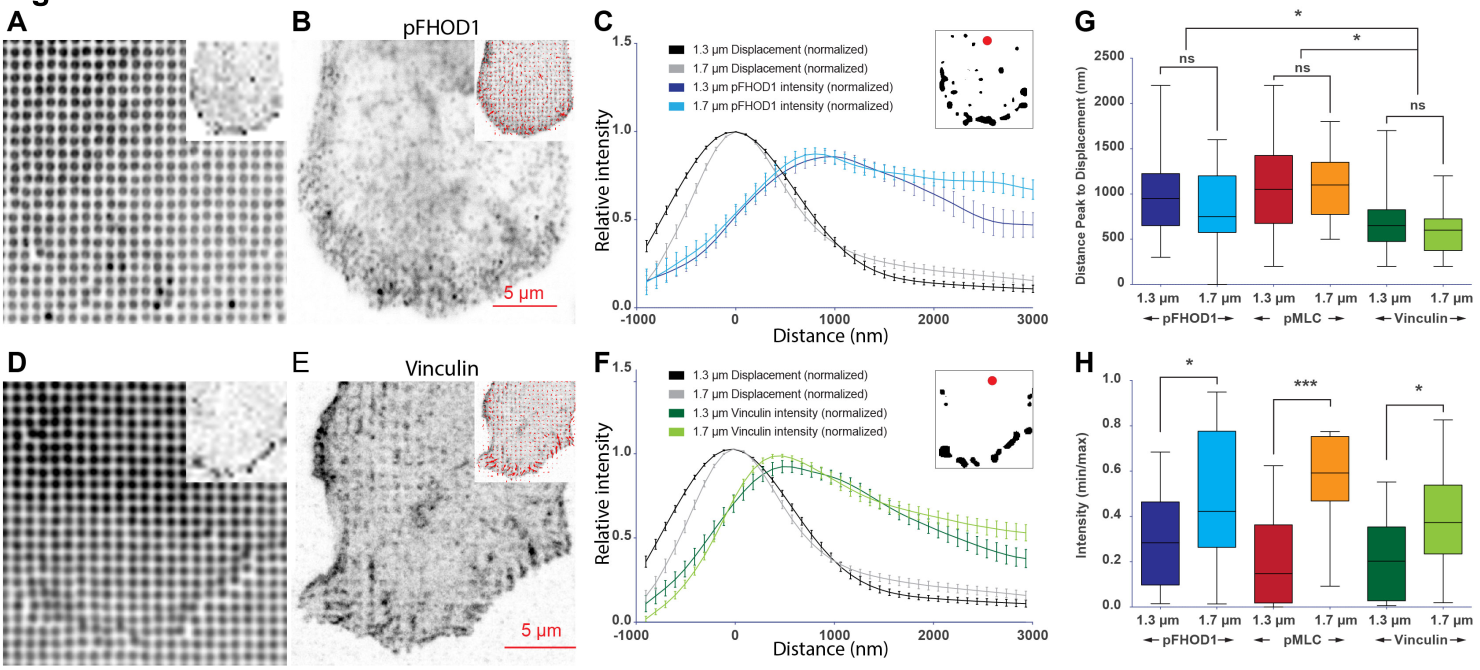

Figure S6: Vinculin and pFHOD1 localize to sites of large pillar displacements. A-F) Freshly isolated NRC were cultured on quantum dot labelled PDMS pillars (A,D, inset shows the grey scale coded displacement map) for 48 hours, fixed and stained for pFHOD1 (B), or Vinculin (E); insets show displacement staining with overlaid displacement vectors. C,F) Radial profiles were calculated from displacements that were higher than the 90th percentile (inset, black areas) to the centre of the cell (inset, marked with red dot). G) All three proteins are enriched inwards of displaced pillars, but Vinculin peaks closer to the displaced pillars. For pMLC, see also Figure S1. H) pFHOD1, pMLC (see Figure S1) and Vinculin are more strongly enriched at force transduction sites on 1.3 μm than on 1.7 μm pillars. Error bars: SEM; Box plots: Tukey; n>12 cells for all conditions. * p<0.05; ** p<0.01; *** p<0.001; **** p<0.0001; ns: not significant; P values from ANOVA and Tukey correction for multiple comparisons. Related to Figure 3.

Figure S7:

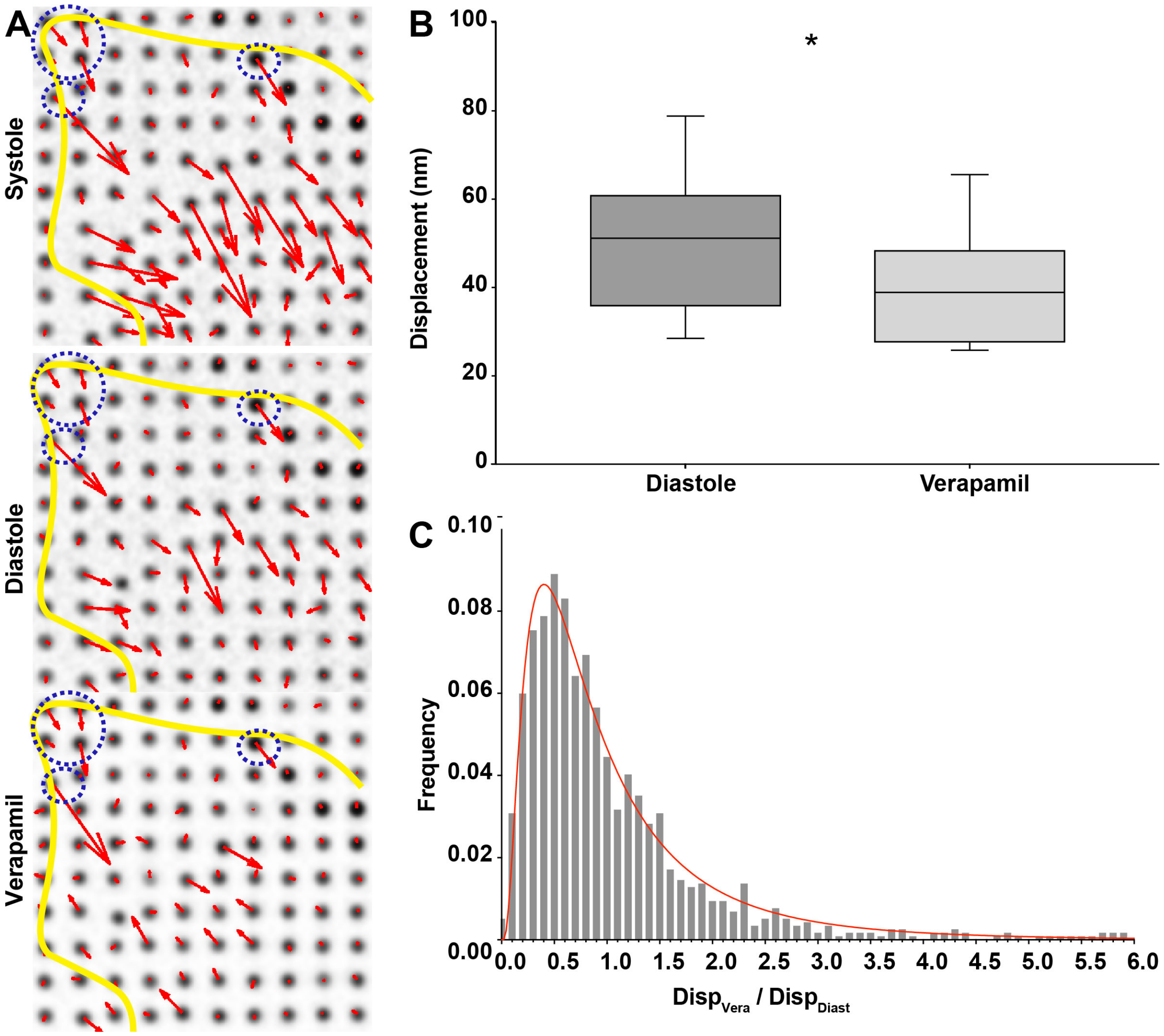

Figure S7: Comparison of diastolic resting tension and residual tension after verapamil treatment. A) Freshly isolated NRC were cultured on quantum dot labelled PDMS pillars (1.3 µm height, k=6pN/nm). Movies were recorded of contracting cardiomyocytes to extract pillar positions during systole and diastole (A), before treatment with verapamil (10µM) for 10 minutes. Further images of the cell were taken after visual confirmation that contractions were blocked (A, Verapamil), and after trypsinisation to recover the original pillar positions. B) Comparison of average pillar displacements of n=12 cells per condition shows a 15% reduction after verapamil treatment (*: p=0.03 from a ratio paired t-test). C) Pillar by pillar analysis of changes to the displacement, combined for all 12 cells. The ratio of the displacement after verapamil treatment vs the displacement during diastole shows a lognormal distribution, peaking around 0.6, i.e. a 40% loss of tension. Related to Figure 3.

Figure S8

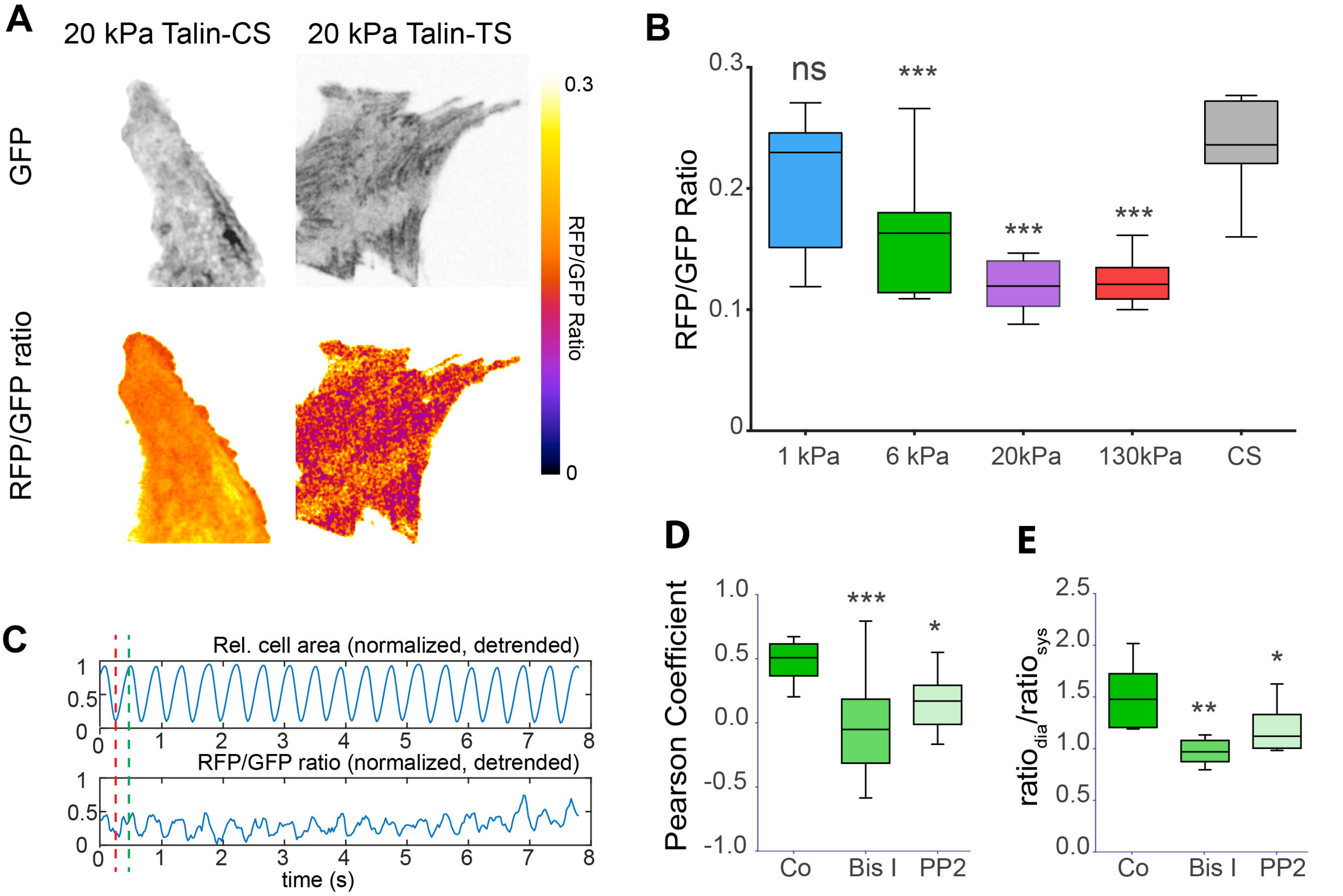

Figure S8: The talin tension sensor indicates higher tension on stiff surfaces in fixed cells. A-B) Freshly isolated NRC, transfected with the Talin tension sensor (TS) or control sensor (CS) were cultured on PDMS coated coverslips for 48 hours, stained and imaged on a ZEISS LSM 880 microscope with spectral detector after illumination with a 488nm laser. The RFP to GFP ratio was calculated after linear unmixing. C) Live cells were imaged with 40 frames per second. The cell edge position was tracked after thresholding and compared to the RFP/GFP ratio at adhesion sites (see Figure 4B), after normalization and detrending to remove changes due to differential bleaching of the fluorophores. Example traces for the cell area and ratio are shown in (C). The Pearson correlation coefficient was calculated (summarized in Figure 4C) and minima (red line) as well as maxima (green line) were identified in the cell area plot and ratio values were averaged over all minima or maxima, respectively (summarized in Figure 4D). Treatment with Bis I or PP2 reduces both Pearson coefficient (D) and the FRET ratio between diastole and systole (E). N=12 cells; Box plot: Tukey; * $p<0.05$; ** $p<0.01$; *** $p<0.001$; **** $p<0.0001$; ns: not significant; P values from ANOVA and Tukey correction for multiple comparisons. Related to Figure 4.

Figure S9

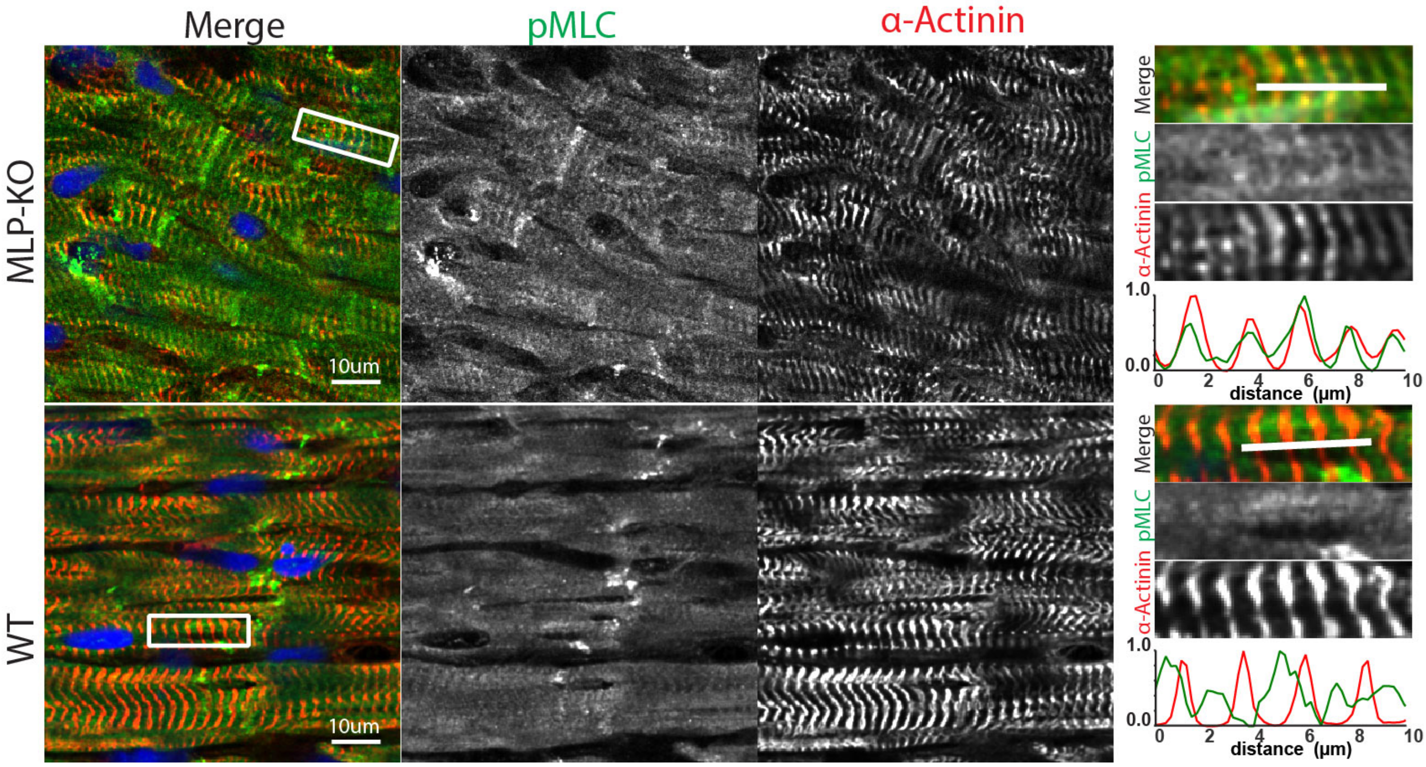

Figure S9: Elevated non-muscle myosin activity in dilate cardiomyopathy. pMLC is found in striations that partially overlap with α-actinin in hearts of the MLP-KO mouse, but not in wild type controls. Related to Figure 5.
